# Improving physical activity behaviors, physical fitness, cardiometabolic and mental health in adolescents - *ActTeens Program*: A protocol for a randomized controlled trial

**DOI:** 10.1371/journal.pone.0272629

**Published:** 2022-08-09

**Authors:** Antonio Stabelini Neto, Géssika Castilho dos Santos, Jadson Marcio da Silva, Renan Camargo Correa, Lorena B. F. da Mata, Rodrigo de O. Barbosa, Anderson Zampier Ulbrich, Sarah G. Kennedy, David R. Lubans

**Affiliations:** 1 Health Science Center, Universidade Estadual do Norte do Paraná, Jacarezinho, PR, Brazil; 2 Post-Graduate Program in Physical Education Associate UEM/UEL, Universidade Estadual de Londrina, Londrina, PR, Brazil; 3 Health Sciences Sector, Department of Integrated Medicine, Research Group in Exercise Medicine (MedEx), Universidade Federal do Paraná, Curitiba, PR, Brazil; 4 Health and Physical Education, School of Health Sciences, Western Sydney University, Kingswood, NSW, Australia; 5 Centre for Active Living and Learning, College of Human and Social Futures, University of Newcastle, Callaghan, New South Wales, Australia; 6 Hunter Medical Research Institute, New Lambton Heights, NSW, Australia; University of Pavia: Universita degli Studi di Pavia, ITALY

## Abstract

This trial aims to investigate the effects of the ActTeens physical activity program, on adolescents’ physical activity level, health-related fitness, cardiometabolic and mental health. The trial will aim to recruit ~140 adolescents (aged 13–14 years). Participants will be randomized into either intervention or control groups. The intervention will be guided by social cognitive theory and self-determination theory and implemented over one school term (24-weeks). The *ActTeens Program* will include: (1) structured physical activity sessions delivered within physical education, including movement-based games and dynamic stretching warm-ups; resistance training skill development; high-intensity training workouts; and cool-downs; (2) self-monitoring plus goal setting for physical activity by pedometer-smart wearable; and (3) healthy lifestyle guidance (social support) by WhatsApp® messages about healthy eating and regular physical activity for the intervention and parents groups. Study outcomes will be assessed at baseline, 24-weeks from baseline, and 12-months from baseline. Physical activity (accelerometer) is the primary outcome. Secondary outcomes include muscular and cardiorespiratory fitness, cardiometabolic profile, and mental health. A process evaluation will be conducted (i.e., recruitment, retention, attendance, and program satisfaction). This project will have the potential to address many questions and debates regarding the implementation of physical activity interventions in low-and- middle-income countries.

**Trial registration:** ClinicalTrials.gov NCT05070377. Registered on 7 October 2021.

## Introduction

Participation in regular physical activity (PA) is associated with numerous health benefits, including improved cardiorespiratory fitness (CRF), body composition, cardiometabolic profile, and mental health [1, 2]. To achieve these health benefits, current national [3] and international [2] guidelines recommend children and adolescents participate in an average of 60 minutes daily of moderate to vigorous physical activity (MVPA) and engage in vigorous physical activity (VPA), muscle-strengthening [e.g., resistance training (RT)] and bone-strengthening activities three days a week. Unfortunately, evidence suggests that 81% of school-aged adolescents (11 to 17 years) across the globe are not meeting these recommendations [4], and in Brazil, only 8.4% of adolescents are sufficiently active [5]. Of additional concern, longitudinal studies have demonstrated that PA and health-related fitness (cardiorespiratory and muscular) levels decline during adolescence [6–8]. According to the Oliveira et al. [9], less than half of the evaluated Brazilian adolescents were classified with adequate level on the physical fitness tests (push-ups and PACER) by Fitnessgram criteria.

Previous studies [10, 11] have found that low CRF and muscular fitness levels are associated with a higher risk of developing various chronic diseases (high blood pressure, hyperglycemia, and dyslipidemia). In this perspective, Roldão da Silva et al., [12] founded an inverse association between the muscular fitness (curl-up and push-up) and CRF with the clustered cardiometabolic risk in Brazilian adolescents.

Furthermore, studies also show that PA [13, 14], CRF [15, 16] and muscular fitness [11] have been associated with mental health outcomes. These findings have important implications regarding the mental health of Brazilian adolescents, since, data from the Erica (Study of Cardiovascular Risks in Adolescents) reported a high prevalence (30.0%) of common mental disorders (symptoms of depression and anxiety) in the young population [17].

Importantly, physical fitness in childhood and adolescence has been considered a powerful predictor of health later in life [18] and adequate levels of PA are essential for the development and maintenance of health-related fitness. Consistent findings showed that a high level of MVPA was associated with better aerobic and muscular fitness in adolescents [19, 20].

The school environment is a context where young people spend much of the day learning about different topics, including health. As such, schools are a suitable setting to provide opportunities and to inform adolescents about the benefits of regular PA [21, 22]. The Physical Education (PE) classes can help adolescents to consolidate active lifestyle habits that will last across the life-span [21]. Moreover, this opportunity may expose adolescents to varying forms of PA that they may not have been exposed to outside of school.

Despite the potential for schools to influence students’ health behaviors, when exploring the existing literature, previous school-based PA interventions targeting adolescents have had mixed success [23, 24]. Borde and colleagues [24] suggest the effects of school-based interventions targeting adolescents have been trivial. Emerging evidence has identified that poor implementation of programs is a major barrier to the success of school-based interventions [25]. According to Beets et al [26], poor implementation is potentially due to program drift and voltage drop as interventions progress from evaluating efficacy and effectiveness to dissemination.

Research [27] has shown that multicomponent interventions (ie, comprehensive school-based PA programs) appear to be more successful than single-component interventions. Another specific component is social support provided by parents and friends, which are factors that influence the PA levels of adolescents and should be considered in adolescent intervention efforts [28, 29]. Moreover, another alternative strategy is to deliver digital programs (eHealth and mHealth), reviews [30, 31] show the potential of eHealth and mHealth interventions for changing adolescents’ activity behaviors in the short term, particularly when integrated with other intervention components (eg, school-based environmental changes).

Therefore, multicomponent interventions including strategies in the school environment, out-of-school, and parental involvement, have been effective to improve the PA levels [32, 33], physical fitness (cardiorespiratory, muscular) [34, 35], well-being [36], and reduction of the sedentary behavior [37, 38]. However, this evidence base was largely drawn from high-income country, with sparse representation of in low-and- middle-income countries (LMICs). Thus, more research that aims to develop, implement, and evaluate adolescent physical activity interventions considering the context-specific of the LMICs is necessary.

In Brazil, school-based programs [33, 39, 40] have been developed with a focus on the promotion of active behavior in teens, however, these programs only included strategies within the school context and with a predominant focus on the aerobic component of youth PA guidelines. Thus, considering PA benefits for health, knowing the need to develop strategies to encourage active behavior both in and out of school, and the scarcity of muscular fitness programs in schools, there is a need to implement PA integrated programs that provide new opportunities for practice in the school setting and promote an active lifestyle for adolescents.

### Study objectives

This paper describes the protocol for a randomized controlled trial testing a PA program- *ActTeens Program*- to improve the PA level, health-related fitness, cardiometabolic and mental health in adolescents.

The aims of the trial are to: 1) to investigate the impact of 24 weeks PA program on the PA level in adolescents; 2) to verify the effects of 24 weeks PA program on the CRF and muscular fitness, metabolic profile and mental health; and 3) to analyse the potential mediators of PA behaviour change. Finally, the ActTeens Program will undergo detailed process evaluation to determine if the program has been implemented as intended and verify possible factors influencing implementation.

## Materials and methods

### Study design

The trial is registered in the protocol of clinical trials of the National Institute of Health clinicaltrials.gov (NCT05070377) on 7 October 2021 and approved by the human research ethics committee of the States University of Northen of Parana, Brazil (n° 4.452.513). The present study was written following Standard Protocol Items: Recommendations for Interventional Trials (SPIRIT) [41], which was aimed to improve the quality of clinical trial (S1 Checklist). The intervention will be evaluated using a two-arm randomized controlled trial with intervention and usual practice control groups. The assessments will be conducted at baseline, after 24-Weeks (primary endpoint) and after 48-Weeks from baseline (secondary endpoint). Baseline data collection will occur in the school term preceding the intervention (March 2022). The intervention will take place from April to October in public schools in Jacarezinho City. Post-test data collection (ie, 6-month follow-up) will start at the end of October and it will continue until midway through November, with the final following assessments (i.e., 12-month follow-up) being completed in April 2023.

### School recruitment and selection

The secondary public schools in Jacarezinho City, including students from 13–14 years (i.e., Grade 8 and 9) will be eligible to participate. Schools will be recruited through a list provided by the Regional Education Center of Jacarezinho, Pr, regarding the 2022 academic year. Then, emails will be sent directly to eligible schools (school principals and Grade 8 and 9 coordinators). Once schools have expressed interest in the study, a member of the research team (project coordinator) will meet with the school representative(s) and explain the study requirements. At this time, each school will be asked to identify if grade 8 and 9 PE teachers are willing to facilitate the delivery of scheduled *ActTeens* sessions during their lessons.

### Participants

Physical Education teachers of the participating Grade 8 and 9 classes, as well deliver the program during the scheduled class time. Eligible participants will be grade 8 or 9 students aged between 13—and 14 years. Parental signed informed written consent will be obtained from students before participation. This consent authorizes the student’s involvement in the evaluation component of the study, as well as the authorized use of their de-identified data. Students with a cardiometabolic disease diagnosed (type 2 diabetes; hypertension) or with a physical or mental condition that would preclude their participation in physical activity program will be excluded from the study (analysis).

### Sample size calculation

The sample size estimation was conducted using G*Power (version 3.1) and based on detecting changes in the primary outcome of PA. Based on previous research, we anticipate the effect size for PA of d = 0.25 (an adjusted for the baseline of 5 min MVPA per day and assuming a standard deviation of MVPA of 17.8 minutes with a correlation of 0.59 between baseline and follow-up) [42]. We adjusted for clustering at the class level using a correction factor of [1 + (m—1) x ICC], where m represents the number of participants per class and ICC refers to the intraclass correlation coefficient for PA, assuming an average class size of 29 participating students, three classes per school and an ICC for PA of 0.034 [43]. Allowing for an expected dropout of 20% at the study endpoint, the required sample size to achieve 85% power with alpha levels set at 0.05 in 174 students (87 in the intervention group and 87 in the control group).

### Blinding and randomisation

**Fig 1:**
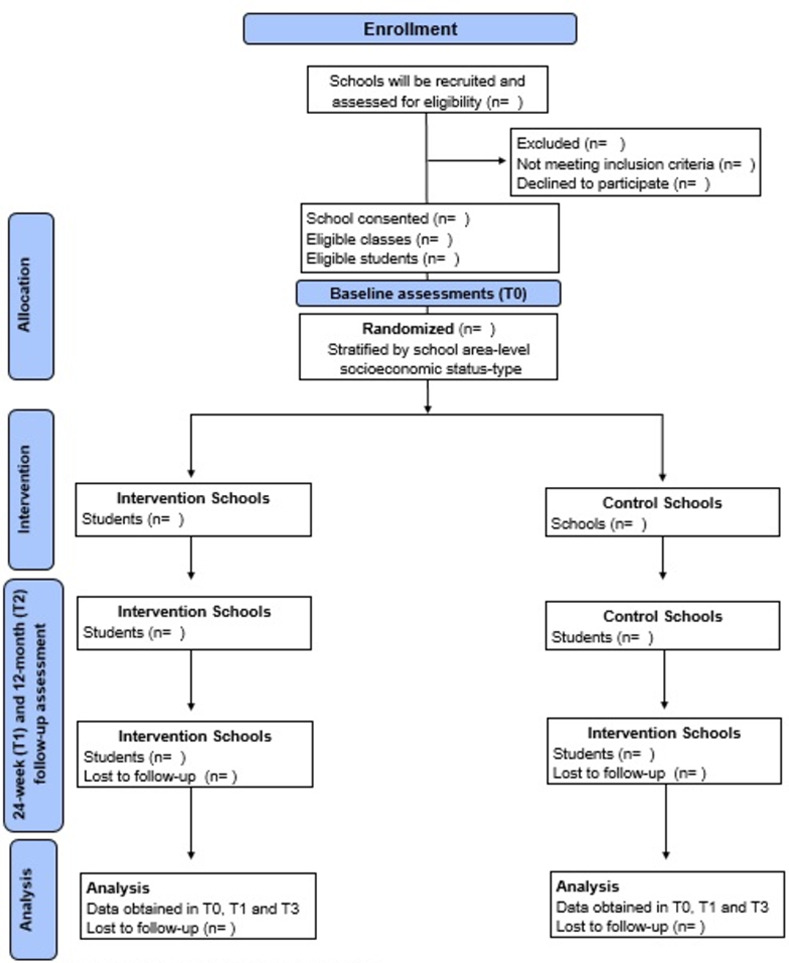
Protocol timeline implemented for the study.

Randomization will occur among schools that have been recruited and have completed baseline assessments. The schools will be matched based on the following characteristics: school area-level socioeconomic status (i.e., using Socio-Economic Class ABEP) [44]. Schools will be randomised to either a control or an intervention condition by an independent researcher using a computer-based random number generator. We will aim to recruit two classes per school (ie, one class of 8 grade and another of 9 grade) (Fig 1). Schools selected to the intervention condition will participate in the program during the study period, whereas schools allocated to the control condition will continue usual school practice.

### Intervention

The ActTeens school-based PA program will be implemented over one school term (16-weeks) and will be designed to improve adolescents’ PA level, fitness, metabolic, and mental health. The *ActTeens Program* will include three major components: (1) structured PA sessions delivered within Physical Education, (2) self-monitoring plus goal setting by a pedometer, and (3) healthy lifestyle guidance (i.e., social support). The program will be guided by social cognitive theory [45] and self-determination theory (SDT) [46] and will target teachers, schools, students, and parents.

The structured PA sessions, which is focused on resistance training (RT) is an adaptation of Resistance Training for Teens [34]. RT for Teens was an Australian program designed to improve muscular fitness and provide adolescents with the knowledge, motivation, skills, and confidence to engage in resistance training. The structured PA sessions have been designed for satisfying participants’ basic psychological needs which include autonomy, competence and relatedness of the students, and so promoting autonomous motivation and self-efficacy for physical activity. The structured PA sessions was adapted from the earlier NEAT [38] and ATLAS programs [47]. The structured PA program will be delivered within PE lessons, twice a week, for 20-minutes per session (40 min·wk^-1^). The implementation strategies used to support adoption and delivery will include: (i) professional learning workshop for teachers; (ii) provision of teacher handbook, session resources, and fitness equipment; and (iii) physical activity session observation and feedback. The program will consist of the following components: (i) an introductory seminar for students delivered by teachers; and (ii) a structured PA program, focused on RT.

The introductory seminar will be designed to explain the rationale for the program and reinforce the importance of exercise for physical health (focus on muscular fitness), metabolic profile, and behavioral change such as physical activity self-monitoring and goal setting [47]. The structured PA sessions [34, 47] will follow a specifics format including i) movement-based games and dynamic stretching warm-up; ii) Resistance training skill development; iii) high-intensity workout, and iv) cool-down. Participants will be able to select from a variety of predesigned circuit cards, which will be released across the program to promote variety and sustain participant interest. The level of intensity for each session component will be guided by Borg´s CR-10 rating of perceived exertion scale.

To promote exercise adherence, sessions will be developed with a focus on enhancing students’ autonomous motivation [34, 47], within and beyond the school setting by satisfying their basic psychological needs for autonomy (feeling in control), competence (feeling capable), and relatedness (feeling connected with others) [46]. Teachers will learn to facilitate sessions using the Supportive, Active, Autonomous, Fair, and Enjoyable (SAAFE) teaching principles [48], which will serve as a framework for the design and delivery of the physical activity sessions, as well as session observations. They will be educated about the importance of and provided with strategies for, integrating SAAFE principles in their lessons during the professional learning workshop.

Participants’ need for autonomy will be satisfied by the teacher-delivery seminar and providing opportunities for choice within sessions (e.g., type of activity and preferred music playing). Competence will be satisfied using positive and specific feedback from teachers to enhance self-efficacy (e.g., providing encouragement, giving specific feedback on technique, modeling correct performance) [47]. Teachers will be encouraged to adopt practices that support relatedness and group cohesion during the sessions (i.e., encouraging supportive behavior among students) [48]. Several precautions will be undertaken to ensure the safety of participants including 1) explanation of correct technique for all exercises at the beginning of the program, whilst students are learning the techniques; 2) inclusion of warm-ups and cool-downs, and 3) reminders for teachers and research staff member to monitor and correct exercise technique.

Regarding the sessions, a range of sociocultural targeting strategies [49] will be applied to the program to increase relevance and appeal to adolescent boys and girls. For example, the circuit cards and interactive seminar will include images of female and male same-sex role models. In addition, the interactive seminar will include content that is relevant to boys and girls, separately and together, by recognizing and focusing on health behaviors common to each sex. However, the sessions will be conducted with mixed-sex groups.

To promote active behavior out of school, a pedometer plus goals setting will be used. Each adolescent within the intervention group will receive the researcher’s personalized goals (based on the number of steps measured in the baseline week), which must be achieved weekly. The goals will be predetermined way progressive [50], from the execution of an additional every two weeks 10% (first and the second week) to 45% (at week 15 and 16), and also will be sent by WhatsApp® messages to encourage adolescents to practice daily PA. Students and their parents will also be sent WhatsApp® messages about healthy eating and regular PA, to work toward improved healthy behaviors [51] (see Table 1 for further details of these strategies).

**Table 1:** Intervention and implementation description.

Component/Strategy	Dose	Description
Professional learning workshop	1x5-h workshop (preprogramme)	The workshop for PE teachers and research staff members will be delivered by principal investigator, which will be addressed all aspects of the intervention: 1) teacher roles and expectations; 2) intervention components; 3) introduction to active behavior, muscle strengthening and safety implications; and 4) philosophy of the programs, including the sex-targeting strategies and explanation of the ‘‘SAAFE” teaching principles.
Support from research team	Once per month	The research team will create an "intervention school" WhatsApp group and invite intervention´s teachers to join. The teachers will be encouraged to share challenges and successful strategies in the app.
Presentation to school staff	1x15 min	The research team will design a tailored presentation to the educational team (i.e., school principals, coordinators and school faculty) during a regularly scheduled staff meeting. The purpose of the presentation is to inform of the objectives and details of the programme, and to promote a supportive school climate.
Provision of teacher handbook, fitness equipment, and session resources, provided by the research team	Once	Schools will be provided facilitator handbooks, sets of circuit cards and fitness equipment packs.
Interactive seminar	1×1 hour seminar	Students will participate in an interactive seminar by a member of the research team. The interactive seminar will provide important information about the programme components and behavioural messages (including current recommendations for youth physical activity, healthy eating, screen-time). It will also address relevant information on physical, metabolic and mental health.
Structured physical activity program	2/week	The session will include bodyweight and elastic tubing resistance training, aerobic-based and strength-based activities, and high-intensity fitness challenges that will be run at school during physical education lessons for 16 weeks (2 times per week). Each exercise session will last approximately 20 min. Students will be able to select from a variety of pre-designed cards incorporating both aerobic-based and resistance-based exercises. Behavioural messages (i.e., be physically active, limit screen time, foods, and sugar-sweetened beverages) will be reinforced at the end of sessions (e.g., cooldown).
Pedometer plus goals setting	16-week	The pedometer plus goals setting will be used as a strategy to promote participation in PA of adolescents (out of school). Participants will wear Yamax SW700 pedometers for physical activity self-monitoring and will receive a new goal-setting (based on the number of steps measured in the baseline week) to achieve weekly. The goals setting will be progressive and changed every two weeks, and this strategy has as its objective propose a realistic goal setting aim to achieve at least 10000–11700 steps/day.
e-health	Twice per week	Adolescents of the intervention group will receive messages about healthy behavior (i.e, active lifestyle and healthy eating) by WhatsApp®.
Parental involvement	Once per week	Parents of intervention group students will receive weekly messages about the benefits of physical activity and healthy eating by WhatsApp® to support their adolescent’s healthy behavior change at home.

Adapted by Resistance Training for Teens program [34]–RT: Resistance training; IG: intervention group.

### Measures and data collection

All assessments will be conducted in the study school by trained research assistants, who will be blinded to group allocation at all time points (baseline, post-intervention, and 12-month follow-up). Socioeconomic information and self-report measures (S3 File) using questionnaires will occur prior to fitness assessment. Anthropometric assessments will be conducted sensitively by same-sex researcher staff when possible. The research assistants will provide a brief verbal description and demonstration of each fitness test before commencement.

### Primary outcome

#### Physical activity

Adolescents will be instructed to wear an Actigraph GT3X accelerometer on the hip at the height of the anterior iliac spine for all days (except when bathing, swimming, sleeping) for seven consecutive days. The subjects who use at least a wear time of >8 h on three days or more (including one weekend) will be included in the valid analyses data. The time (minutes/day) spent in physical activity of different light, moderate and vigorous intensities will be estimated, using validated cut-off points [52]. Non-wear time, defined as ≥60 min of continuous ‘0’ counts, will be removed from the data set [53]. Weekday and weekend days physical activity will be calculated separately (i.e., mean minutes per day).

### Secondary outcomes

#### Muscular fitness

Upper body muscular and lower body muscular endurance will be assessed using a 90-degree push-up [54] and sit-to-stand test [55], respectively. In the 90-degree push-up test, the participant should lower their body until a 90-degree angle is formed at the elbow before pushing back up, using a cadence of 40 beats per minute. The test will be concluded when the participant either fails to do a push-up in the angle required on two non-consecutive repetitions (warning verbalised by an assessor, repetitions counted), fails to maintain movement in time with the metronome, fails to maintain appropriate technique (back straight) or on the volitional failure of the test. To assess lower body muscular endurance, participants will sit in a chair (regardless of the height of the participant) with his/her back against the back of the chair. The students will be asked to go from a sitting to a standing position and back to a sitting position for 30 seconds as many times as possible.

#### Cardiorespiratory fitness

Will be assessed using the PACER FITNESSGRAM test and will be administered following standardized procedures [56], which have excellent validity and reliability in this population [57]. Test administrators will provide verbal encouragement during the shuttle to maximize participant motivation. A 20m course will be set up on a hard surface with participants instructed to run back and forth between two sets of lines while keeping pace with a pre-recorded cadence (indicated by a single beep for each 20m shuttle). The test begins at 8.5km/h (slow pace), and increases by 0.5km/hour with each passing minute. The test will be terminated when the participants fail to complete two consecutive laps in the allotted time or voluntarily dropped out due to fatigue. The last successful stage will be recorded and converted into the number of 20m laps completed, and the total number of laps will be used to estimate maximal aerobic capacity (VO_2max_) using the equation: 45.619+(0.353*PACER laps)–(1.121*age) [58].

#### Cardiometabolic health

The cardiometabolic variables that will be analyzed are glucose, insulin, triglyceride, total cholesterol, and glycated hemoglobin. Blood samples will be collected from the antecubital vein in vacuum tubes after 12 hours of fasting following the recommendations of the Brazilian Society of Clinical Pathology/Laboratory Medicine, at two different moments (baseline and postintervention). Fasting glucose will be measured using the reference enzyme with Hexokinase. To determine fasting insulin, the chemiluminescence method will be used. The total cholesterol and triglycerides will be analyzed by the enzymatic colorimetric method, and glycated hemoglobin will be determined using high-performance liquid chromatography. The homeostasis model assessment for insulin resistance (HOMA-IR) will be used to determine insulin sensitivity, and will be calculated using the following formula: [(glucose* 0,0556)*insulin]/22,5 [59].

#### Mental health

The anxiety, depression, and stress symptoms of the participants will be assessed using the Depression and Anxiety Stress Scale-short form (DASS 21) [60, 61]. This scale comprises 21 questions using a 4-point Likert-type scale (0 = not at all, 3 = almost always), covering three dimensions of mental health status(S3 File). The total score of each dimension will be multiplied by two and scores ranged from 0 to 42.

The well-being will be measured by two domains of the KIDSCREEN-27 questionnaire (S3 File), physical well-being consists of 5 items, and psychological with 7-item. For each item, participants respond using a scale with scores from one to five points [62].

Sleep quality and quantity will be collected by Pittsburgh Sleep Quality Index (PSQI) [63], which is validated for Brazilian adolescents [64]. The PSQI is a self-reported questionnaire(S3 File) that asks respondents to report their sleep quality and signs of sleep disturbance for 1 month before completing the questionnaire. The PSQI includes 19 questions, categorized into seven groups (sleep quality, sleep latency, sleep duration, habitual sleep efficiency, sleep disturbance, use of sleeping medications, and daytime dysfunction). Each constituent question produces a score on a 4-point Likert-type scale (from 0 to 3) and the total score is made up of scores from each of the seven subgroups of questions, giving a cumulated score between 0 and 21.

#### Anthropometric assessments

Body mass will be measured to the nearest 0.1 kg in light clothing without shoes using a portable digital scale (Welmy®, Santa Bárbara do Oeste, São Paulo, Brazil) and height will be recorded using a portable stadiometer (Welmy®, Santa Bárbara do Oeste, São Paulo, Brazil). Both weight and height will be measured twice to reduce the risk of measurement error. A third measurement will occur if there is a difference of >0.1 kg for body mass and >0.3 cm for height between the first and second measurements. Body mass index (BMI) will be calculated using the standard equation (body mass [kg]/height [m]2) and BMI-z scores will be determined using the ‘LMS’ method according to World Health Organization data [65]. Waist circumference will be measured twice at the midpoint between the last rib and the iliac crest using steel tape (Sanny®, São Bernardo do Campo, São Paulo, Brazil). A third measurement will occur if there is there be a difference of >0.3 cm for WC.

#### Healthy lifestyle

Screen time will be estimated by the following question: “during an ordinary weekday, how many hours do you spend watching TV, using a computer, smartphones or playing video games?” (except Saturday, Sunday, and holidays) [66]. Eating behaviors will be assessed by questions about the frequent consumption of ultra-processed/embedded food, sugary foods, snacks, and sugar drinks. Also it will be asked about breakfast by the following question:”Considering a normal week, how many days do you have breakfast?” [67]

### Hypothesized mediators

#### Resistance training self-efficacy

Self-efficacy will be assessed by a four-item scale(S3 File) developed specifically for use with adolescents [68], and the participants will respond about their confidence to engage in resistance training using a 5-points Likert (1 = strongly disagree to 5 = strongly agree).

#### Basic psychological needs satisfaction

The ‘Adolescent Psychological Need Support in Exercise Questionnaire’ will be used to evaluate friends´ and teachers’ support for exercise [69] (S3 File). The evaluation of this instrument requires satisfaction during exercise across the three-item: autonomy support, relatedness support, and, competence support. Participants reported their satisfaction using a 7-point Likert scale ranging from *1 ‘strongly disagree’ to 7 ‘strongly agree’*.

#### Autonomous motivation

The ’Behavioural Regulations in Exercise Questionnaire’ [70] (S3 File) will be used to assess autonomous motivation for physical activity using 2-subscales: identified and intrinsic regulations. Adolescents respond on a five-point scale ranging *from 0 ‘not true for me’ to 4 ‘very true for me’*.

### Control variables

#### Somatic maturation

Biological maturation will be estimated through the evaluation of somatic maturation by determining the distance in years of the individual from the baseline peak height velocity (PHV) using sex-specific mathematical models [71]. This method estimates maturity-offset (in years) from height and chronological age using Eq 1 for boys:

$$\text{Maturity}\;\text{offset}\;\text{for}\;\text{boys}\;\left(\text{years}\right)=-7.999994+\left(0.0036124\times \left(\text{age}\times \text{height}\right)\right)$$ (1)

And using the Eq 2 for girls:

$$\text{Maturity}\;\text{offset}\;\text{for}\;\text{girls}\;\left(\text{years}\right)=-7.709133+\left(0.0042232\times \left(\text{age}\times \text{height}\right)\right)$$ (2)

#### Lifestyle questionnaire

Information on alcohol drinks and tobacco smoking(S3 File) among adolescents will be obtained by two questions: “Do you smoke?” and “Do you alcohol beverage?” in a dichotomous manner (yes or no), through indication of the use during the previous 30 days.

### Process evaluation

Feasibility will be assessed based on the following: 1) consent rate (how many participants offered the program agreed to be involved); 2) retention rate (retention rate at 24-weeks follow-p); 3) attendance (student participation in the structured PA), 4) students´ satisfaction with the program (“I enjoyed participating in the sessions on 5-point Likert scale: 5 = strongly agree to 1 = strongly disagree) (S4 File), 5) engagement (student engagement with the pedometer self-monitoring and adherence to goals setting) and 6) practical session fidelity (three observations per teacher) using the SAAFE observation checklist [48] (S4 File).

### Safety considerations

All measurements, evaluations, and interventions in the context of the present study will be performed in a safe environment, however, if any adverse event occurs, the research team will call the Mobile Emergency Care Service. In addition, adolescents’ vital signs (blood pressure, heart, and respiratory rate) will be continuously monitored during measurements and intervention (if need). All adverse events will be evaluated, recorded, and discussed in the final paper.

### Statistical analyses

#### Analysis plan

**Fig 2:**
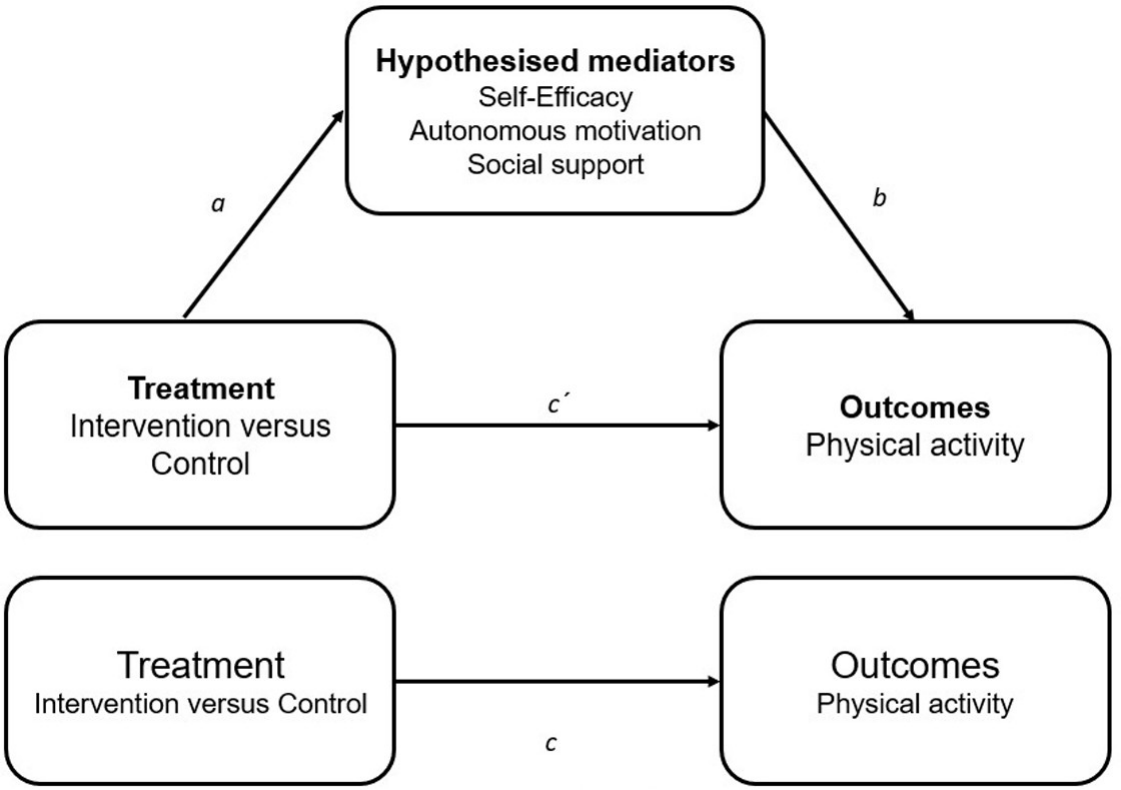
Hypothesized mediators of physical activity behavior change.

Linear mixed models will be used to analyze the primary and secondary outcomes using IBM SPSS Statistics for Windows (version 20.0; 2010 SPSS Inc, IBM Company, Armonk, NY 2010 SPSS Inc., IBM Company, Armonk, NY), with significance set at p< 0.05. All analyses will subscribe to the intention-to-treat principle, whereby all participants will be included in the analysis in the group to which they were randomized. The models will be used to assess the effect of treatment, time and the group-by-time interaction, using random effects to account for the clustered nature of the data. Time and treatment will be included as fixed factors, with school class will be included as a random effect. According previous studies, school-level clustering is negligible after accounting for clustering at the class level [72]. However, if requerid, we will test this assumption and additionally adjust our analyses for school-level clustering. Mixed models are consistent with the intention-to-treat principle, assuming that data are missing at random. The analyses of the primary endpoint (ie, 24 weeks endpoint) will be performed using a repeated measures analysis of covariance applied using mixed linear models. Change from baseline to the postintervention of primary and secondary outcomes will be applied as the dependent variable, treatment (intervention or control), time (baseline and 24 weeks), and the group-by-time interaction will be included as independent variables. To examine the maintenance of the effect of the physical activity program on primary and secondary outcomes, comparable repeated measures approach will be used between group (intervention or control), time (baseline, 24 weeks and 48 weeks), and group-by-time interaction. Effect sizes between groups will be calculated using Cohen’s *d* (the adjusted difference between the control and intervention groups over time divided by the pooled standard deviation of change) and interpreted as follows: *d* = 0.2 (small), *d* = 0.5 (medium), and *d* = 0.8 (large). Potential moderators will be explored using linear mixed models with interaction terms for the following: sex (male, female), socioeconomic status (low, medium, high), and initial BMI status (healthy weight vs overweight/obese). Subgroup analyses will be only conducted if significant interaction effects P≤0.10. Sensitivity analyses will be performed (eg, multiple imputation and complete-case analysis). If the intervention effect estimate differs differs significantly from that obtained when these values are included, both results will be reported in the paper. For completeness in assessing the effect of missingness, we will also conduct one per-protocol analyses (ie, at the student levels). Therefore, our student-level per-protocol analysis, we will include only those students who participated at least 80% of sessions offered across the intervention period (up until our primary end point). Hypothesized mediators (Fig 2) of physical activity behavior change will be examined using structural equation modelling to assess the underlying causal pathways of the intervention.

### Data management

All data will be entered into the database using unique study codes for each participant and will be securely stored on a password-protected computer. The data manager, who is independent of competing interests, can only access the data. A Data Monitoring Committee is not needed since the study is minimal risk. Important protocol modifications during this study will be regularly communicated and updated on the Clinical Trial Registry and the journal of publication. No later than three years after collecting the 48-Weeks end-line assessment from baseline, we will deliver a wholly without identified data set to an appropriate data archive for sharing purposes.

### Patient and public involvement

The study will be conducted in secondary public schools in Jacarezinho and participants (i.e., students and teachers) will be invited to provide feedback on the intervention and suggestions for further improvement, which will be used to refine the ActTeens Program intervention components and implementation strategies. The findings of this trial will be published in peer-reviewed journals, irrespective of the direction or magnitude of the results, and will also be presented both to the Regional Education Center of Jacarezinho and participating schools through a report outlining the findings.

## Discussion

It is widely acknowledged that the health benefits of participation in PA are not limited to physical health but also incorporate metabolic and mental components [2]. Despite the importance of PA, data from the Brazilian Scholar Health Survey (PeNSE in Portuguese) found that the prevalence of Brazilian adolescents who met the PA guidelines was extremely low [5]. The *ActTeens* trial will investigate the impact of a 16-weeks of PA program on physical activity levels, fitness, cardiometabolic and mental health in adolescents. The findings of this trial will contribute to the growing evidence around PA interventions in low-and- middle-income countries, which are still scarce, particularly in South America.

The *ActTeens Program* (adaptation of the Australian RT for Teens program) [34] aims to provide students with additional PA opportunities within the school day, to increase fitness and physical activity levels. It also includes self-monitoring, individualized goal-setting, and parent involvement to promote active behavior out-of-school. Strategies included within ActTeens will be guided by self-determination theory and social cognitive theory, which will endeavor to satisfy students’ basic psychological needs (autonomy, competence, and relatedness) and promote self-efficacy, respectively. The hypothesis is that the intervention will effectively be improving fitness levels, cardiometabolic and mental health, and increase the physical activity levels of adolescents.

The structured PA sessions will be specifically designed to provide adolescents with the knowledge, skills, and new opportunities to engage in PA through resistance training focusing on enhancing muscular fitness. Evidence that additional resistance training during PE classes has a positive impact on screen-time [37, 38], body composition [73], muscular fitness [34, 37, 73], resistance training skill competence [34, 37], self-efficacy [34], autonomous motivation for PA [34], and well-being [36]. The structured sessions of ActTeens Program could offer a feasible method to improve CRF and muscular fitness within PE classes. Further potential advantages are its scalability and ease of dissemination [47, 74] since this intervention may be incorporated into PE lessons without taking up additional PE time; it should not interfere with the normal curriculum and does not require extensive training of teachers.

To address students’ PA levels throughout the entire day and promote PA outside of school, the program will include behavior change strategies such as self-monitoring and individualized goal-setting through pedometer use, and parent involvement (social support). The pedometer will be strategies that might have a large potential to motivate the PA practice daily, since adolescents have the freedom to choose when, how, and with whom he/she wants to be physically active to reach/ increase the steps/day [32]. The parents will play as encouragers for their children to be physically active in the home environment since social support is a determining and have a pivotal role in behavioral change and adherence to a healthy lifestyle.

The strengths of the ActTeens Program include: 1) a new PA opportunity different from the traditional activities that have been focused for PE lessons; 2) individualized goal-setting of steps/days; 3) autonomy of adolescents to choose which exercise/activities to do both during structures sessions and out-of-school to achieve the goal. Furthermore, all participants will be followed up for 12 months after the intervention period. A long follow-up period is clinically relevant but has not been previously investigated in Brazilian adolescents. Extended follow-up will provide information on maintenance of health effects and long-term outcomes (eg, effects on metabolic markers).

In conclusion, the current study will provide evidence regarding the impact of a PA intervention on fitness, active lifestyle, cardiometabolic and mental health over the short and long-term in adolescents, with potential to debates about design and implement of PA interventions in low-and- middle-income countries.

## Supporting information

S1 Checklist SPIRIT checklist. (DOC)

S1 File Institute’s ethics committee protocol–version in English. (DOCX)

S2 File Institute’s ethics committee protocol–version in Portuguese (original). (DOCX)

S3 File ActTeens questionnaire. (DOCX)

S4 File ActTeens_ process evaluation. (PDF)
