# Supplementary material for: Improving physical activity behaviors, physical fitness, cardiometabolic and mental health in adolescents - ActTeens Program: A protocol for a randomized controlled trial
Source: PLoS One. 2022 Aug 9;17(8):e0272629. doi: 10.1371/journal.pone.0272629 (PMC9362910; doi:10.1371/journal.pone.0272629)
Supplement: S1 File — (DOCX) [file pone.0272629.s002.docx]

**Effects of a physical activity promotion program on the level of physical activity, physical fitness, cardiometabolic and mental health in adolescents**

**Introduction**

The practice of regular physical activity (PA) has been associated with numerous health benefits including improve cardiorespiratory fitness (CRF), body composition, cardiometabolic profile and mental health ([1](#_ENREF_1), [2](#_ENREF_2)). In order to achieve these health benefits, several national and international organizations/health authorities have issued guidelines that recommend children and adolescents participate in at least of 60 min daily of moderate to vigorous physical activity (MVPA) and engage in muscle-strengthening activities [e.g., resistance training (RT)] 3 days a week ([2](#_ENREF_2)). Furthermore, evidence suggests that 81% of school-aged adolescents aged 11 to 17 years not meeting the current recommendations([3](#_ENREF_3)), and in Brazil, only 8.4% adolescents are physically active ([4](#_ENREF_4)). Of additional concern, longitudinal studies have demonstrated that physical activity and health-related fitness (cardiorespiratory and muscular) level decline during adolescence ([5-7](#_ENREF_5)), both of which are important predictors of health status.

Previous evidence ([8](#_ENREF_8), [9](#_ENREF_9)) observed that low levels of cardiorespiratory fitness and muscular strength are related to higher risk factors for chronic disease. Importantly, physical fitness in childhood and adolescence has been considered as a powerful predictor of health later in life([10](#_ENREF_10)) and adequate levels of physical activity (PA) being essential to develop and maintain a healthy level of physical fitness. Consistent findings showed that a high level of moderate-vigorous PA (MVPA) was associated with better aerobic and muscular fitness in adolescents ([11](#_ENREF_11), [12](#_ENREF_12)). Thus, interventions focused on promoting active and improve physical fitness in adolescents are a public health priority.

The school environment is a context where young people spend much of the day assimilating knowledge about different topics, including health. In this way, schools are an ideal setting to provide opportunities for inform adolescents about the benefits of regular PA ([13](#_ENREF_13), [14](#_ENREF_14)). In addition to guiding and educating young people about this behaviour, schools play a key role in providing structured and unstructured PA ([14](#_ENREF_14)). One opportunity for this to happen is through physical education (PE) classes that represent the ideal medium for the education and overall training of adolescents, which helps them to consolidate active lifestyle habits that will last throughout the rest of their lives([13](#_ENREF_13)). Moreover, this opportunity may expose adolescents to varying forms of PA that they may not have been exposed to outside of school.

Studies have shown that adolescents who have higher frequency of participation in PE classes are more likely to spend more time in moderate-to-vigorous-intensity physical activity (MVPA) and less time sitting throughout the day([15](#_ENREF_15)), to have better academic performance([16](#_ENREF_16)), greater social interactions and lower cardiovascular risk([17](#_ENREF_17)) compared to peers who do not attend PE classes. However, recent systematic review and meta-analysis([18](#_ENREF_18)) investigating time spent in MVPA during school PE lessons suggests that secondary school students only spend on average 40.5% of lesson time participating in MVPA. In relation to PE time MVPA, Brazilian adolescents had a low proportion of time (16%) spent on this behaviour([19](#_ENREF_19)). One of the explanations for this observation may be the fact that PE has focused remains solely on traditional, competitive team sports and games([20](#_ENREF_20)) which may not be an effective way and enjoyable for some young people to engage in MVPA.

Despite the potential for schools to influence students’ health behaviours when exploring the existing in the literature, previous school-based physical activity interventions targeting adolescents have had mixed success([21](#_ENREF_21), [22](#_ENREF_22)). One possible explanation in regard to this finding can be due to researchers not consider key factors in the elaborate of intervention, such as factors that facilitate (facilitators) and hamper (barriers) to adolescent participation in PA ([23](#_ENREF_23)). Like this, to develop an effective school–based intervention is needed to consider factors that influence adolescent PA as enjoyment, perceived competence, motivation, social support (teachers, friends and parents), accessibility and availability of new PA opportunities ([23](#_ENREF_23), [24](#_ENREF_24)).

However, few studies of interventions are approach new opportunities (non-traditional activities). In addition, global data suggesting that adolescents have identified a desire to try a variety in physical activities such as weight training and fitness classes ([25](#_ENREF_25), [26](#_ENREF_26)). Of note, these activities become more popular with increasing age and can contribute substantially to adult physical activity([26](#_ENREF_26)).

Given the capacity for schools to provide students with the skills and confidence to engage in lifelong physical activities([25](#_ENREF_25)) and considering the lack of emphasis on alternative physical activities in school–based interventions/program, there is need to introduce activities commonly practiced across the life-span, such as resistance training. From this perspective, fitness activities appear to be a suitable mediator to meet the minimum recommendation of daily PA.

In general, the youth PA interventions described in research predominantly focus on the aerobic component of the youth PA guidelines, with very few focusing on muscular fitness (MF). In response to these limitations, some effective interventions ([27-30](#_ENREF_27)) including RT in adolescents have been conducted in the school setting. Briefly, the intervention resulted in reduced screen-time([27](#_ENREF_27), [29](#_ENREF_29)) and improved body composition([28](#_ENREF_28)), muscular fitness([28-30](#_ENREF_28)), resistance training skill competence([29](#_ENREF_29), [30](#_ENREF_30)) and self-efficacy([30](#_ENREF_30)), autonomous motivation for physical activity([30](#_ENREF_30)), and well-being([31](#_ENREF_31)). However, a number limit of physical activity programs in the school setting is being conducted in low-and- middle-income countries, such as Brazil.

In Brazil, school-based programs ([32-34](#_ENREF_32)) have been developed with a focus on the promotion of active behaviour but none program had as target physical fitness. Thus, considering that (i) adolescence is a period of biological and social transition, in which future patterns of adult health are established, (ii) the benefits of PA and its decline among adolescents, (iii) the important role of muscular fitness for health, and (iv) schools are ideal setting to promote new physical activity practice opportunity for adolescents, there is a need to develop school-based program with focus on MF through of muscle-strengthening activities.

**Study aims**

Primary aim:

- Verify the effect of the school physical activity program on the muscle fitness of adolescents.

Secondary aims:

- Evaluate the feasibility and preliminary efficacy of the program to improve health-related fitness, cardiometabolic health and increase physical activity level in a sample of adolescents;

- Analyse potential mediators of physical activity behaviour change.

**METHODS**

**Study Design**

This project is a randomized clinical trial with during 16 weeks, multiple components to promote physical activity for adolescents, and the design, conduct, and reporting will adhere to the guidelines of the Consolidated Standards of Reporting Trials ([35](#_ENREF_35)). This study will start in the first semester of 2022 in the city of Jacarezinho, Paraná, and data collection will take place at three different times: baseline (February 2022) and post-intervention (June 2022) and follow-up (February of 2023).

**Population, school recruitment and selection**

The secondary public schools in Jacarezinho, including students from 13 – 14 years (i.e., Grade 8 and 9) will be eligible to participate. Schools will be recruited via the list provided by the Regional Education Center of Jacarezinho, Pr, regarding the 2022 academic year. Then emails will be sent directly to eligible schools (school principals and Grade 8 and 9 coordinators). Once schools have expressed an interest in the study, our project coordinator will meet with the school representative(s) and explain the study requirements. At this time, each school should provide a list with the number of classes, for Grade 8 and 9, one class of each year education will be randomly selected to participate. There are no restrictions regarding the teaching discipline (e.g., physical education, mathematics, Portuguese).

Two grades 8 and 9 (one each year) teachers per school that agree to participate in the program during the scheduled class time. Eligible participants will be grade 8 or 9 students who are taught by one of the “Intervention School”. It is important to note that students who agree to participate in the trial are consenting to the evaluation component (i.e., completion of study measures). Prior to the assessments, the parents or legal guardians of the adolescents who accept to participate completed and signed an informed consent authorization the use of their data (Annex I and II). Students with the cardiometabolic disease diagnosed (type 2 diabetes; hypertension) and with a physical or mental condition that would preclude their participation in physical activity program will be excluded from the study (analysis), but will still participate in normal lessons.

**Sample size calculation**

The sample size estimation was conducted using G*Power (version 3.1) and based on detecting changes in the primary outcome of physical activity. Based on previous research, we anticipate the effect size for PA of d = 0.25 (an adjusted for baseline of 5 min MVPA per day and assuming a standard deviation of MVPA of 17.8 minutes with correlation of 0.59 between baseline and follow-up)([36](#_ENREF_36)). We adjusted for clustering at the class level using a correction factor of [1 + (m - 1) x ICC], where m represents the number of participants per class and ICC refers to the intraclass correlation coefficient for PA. Assuming an average class size of 29 participating students, three classes per school and an ICC for PA of 0.034([37](#_ENREF_37)). Allowing for an expected dropout of 20% at the study endpoint, the required sample size to achieve 85% power with alpha levels set at 0.05 is 174 students (87 in intervention group and 87 in control group).

The estimated sample consisted of 174 adolescents of both sexes (87 in the intervention group (IG) and 87 in the control group (CG)) who met the following inclusion criteria: (I) signed informed written consent; (II) be regularly enrolled in the 8th or 9th grade of elementary school II; (III) age between 13 and 14 years. The study exclusion criteria were: (I) having recognized cardiometabolic disease; (II) have any type of genetic, neoplastic or mental illness that could interfere with the understanding of the activity or performance during the research; (III) not having participated in all stages of the study. It is important to emphasize that adolescents diagnosed with cardiometabolic diseases (type 2 diabetes; hypertension) and with a physical and mental condition that may interfere with their participation in the program were excluded from the study analyses, but still participated in the classes and intervention normally.

**Blinding and Randomisation**

Randomization will occur among schools that have been recruited and have completed baseline assessments. The schools will be matched based on the following characteristics: school area-level socioeconomic status (i.e., using Socio-Economic Class ABEP)([38](#_ENREF_38)). Schools will be randomised to either a control or an intervention condition by independent research using a computer-based random number generator. Schools randomised to the intervention condition will the program during the study period, whereas schools allocated to the control condition will continue usual school practice (normal curricular lessons) for the duration of the study period intervention (4-month from baseline). And after the final study assessment, schools allocated to the control group will then receive the intervention.

**INTERVENTION**

The program is a school-based RT intervention, it is an adaptation of the Australian Resistance Training for Teens Program([30](#_ENREF_30)), which originated from NEAT and ATLAS interventions([39](#_ENREF_39)), and will be designed to improve adolescent´s physical activity level, physics, and metabolic health. This multicomponent program will be developed over one school term (16 wk) for approximately 90 min·wk ^-1^ and will target teachers, schools, students and parents. The implementation strategies used to support adoption and delivery will include: (i) professional learning workshop for teachers; (ii) provision of teacher handbook, session resources and fitness equipment; and (iii) physical activity session observation and feedback. The intervention will consist of the following components: (i) introductory seminar for students delivered by teachers; (ii) a structured physical activity program, which focused on RT by circuit cards (physical education); (iii) pedometer for physical activity self-monitoring plus goal-setting (out-of-school); (iv) messages for students and their parents. A summary of the intervention components is provided in table 1.

The intervention will be guided by social cognitive theory (SCT)([40](#_ENREF_40)) and self-determination theory (SDT)([41](#_ENREF_41)), sessions will be designed to satisfy participants’ basic psychological needs for autonomy, competence, and relatedness, to support autonomous motivation and self-efficacy for physical activity. A range of sociocultural targeting strategies([42](#_ENREF_42)) will be applied to the interventions to increase their relevance and appeal to adolescent boys and girls. For example, the circuit cards and interactive seminar will include images of females and males-sex role models. In addition, the content of the interactive seminar will be designed to be relevant to boys and girls by recognising and focusing on health behaviors common to each sex. However, the sessions will be conducted with mixed-sex groups.

The structured physical activity of resistance training([30](#_ENREF_30), [39](#_ENREF_39)) will follow specific session format, including: movement-based games and dynamic stretching warm-up; RT skill development; high intensity RT (HIRT) workout; modified game involving fitness infusion, boxing, or core strength activity; and static stretching. Participants will be able to select from a variety of predesigned RT circuit cards, which will be released across the program to promote variety and sustain participant interest. The level of intensity for each session component will be guided by Borg´s rating of perceived exertion scale.

To promote exercise adherence, physical activity sessions will be developed with a focus on enhancing students’ autonomous motivation for RT([30](#_ENREF_30), [39](#_ENREF_39)) within and beyond the school setting by satisfying their basic psychological needs for autonomy (feeling in control), competence (feeling capable) and relatedness (feeling connected with others) founded upon the tenets of SDT([41](#_ENREF_41)). Teachers will learn to facilitate RT sessions using the Supportive, Active, Autonomous, Fair, and Enjoyable (SAAFE) teaching principles([43](#_ENREF_43)), which will serve as a framework for design and delivery of the physical activity sessions, as well as observations sessions. They will be educated about the importance of, and provide with strategies of, integrating SAAFE principles in their lessons by learning workshop.

Participants’ need for autonomy will be satisfied by providing opportunities for choice within sessions (e.g., type of activity and preferred music playing) and explaining the rationale for the program in an information seminar. The introductory seminar will reinforce the importance of exercise for physical health (focus in muscular fitness), metabolic profile and behavioural change such as physical activity self-monitoring and goal setting. Competence will be satisfied using positive and specific feedback from teachers to enhance self-efficacy (e.g., providing encouragement, giving specific feedback on technique, modelling correct performance)([39](#_ENREF_39)). Teachers will be encouraged to adopt practices that support relatedness and group cohesion during RT sessions (i.e., encouraging supportive behaviour among students)([43](#_ENREF_43)). Several precautions will be undertaken to ensure the safety of participants including: 1) explanation of correct technique for all exercises in the introductory student seminar session; 2) inclusion of warm-ups and cooldowns; and 3) reminders for teachers and research staff member to monitor and correct exercise technique.

Additional strategies used will be a pedometer plus goals setting where each adolescent of the intervention group will receive their own goal (based on the number of steps measured in the baseline week) outlining the goals to achieve weekly. The goals will be predetermined by the researcher of way progressive([44](#_ENREF_44)), and also will be sent by WhatsApp® messages to support healthy eating and regular PA for the intervention, control, and parents ([45](#_ENREF_45)) groups. The control group participated in usual practice (regularly scheduled PE and cocurricular school sport) for the duration of the intervention and received the intervention after the 4-month assessments.

**Measures and data collection**

All assessments will be conducted at the study school by trained research assistants, who will be blinded to group allocation at all time-points. Socioeconomic (SES) information and self-report measures using questionnaires and will occur prior to fitness assessment. Anthropometric assessments will be conducted in a sensitive manner by same-sex researcher staff when possible. The research assistants will provide a brief verbal description and demonstration of each fitness test prior to commencement.

**Primary outcome**

Physical activity

Adolescents will be instructed to wear an Actigraph GT3X accelerometer on the hip at the height of the anterior iliac spine for all day (except when bathing, swimming, sleeping) for a period of seven consecutive days. The subjects who use at least wear time of >8 h on 3 days or more (including 1 weekend) will be included in the valid analyses data. The time (minutes/day) spent in physical activity of different light, moderate and vigorous intensities will be estimated, using validated cut off points([46](#_ENREF_46)). Non-wear time, defined as ≥30 min of continuous ‘0’ counts, will be removed from the dataset ([47](#_ENREF_47)). Weekday and weekend days physical activity will be calculated separately (i.e., mean minutes per day).

**Secondary outcomes**

Muscular fitness

Upper body muscular and lower body muscular endurance will be assessed using a 90-degree push-up ([48](#_ENREF_48)) and sit-to-stand test ([49](#_ENREF_49)), respectively. In the 90-degree push-up test, the participant should do push-up until a 90-degree angle is formed at the elbow before pushing back up, using a cadence of 40 beats per minute. The test will be concluded when the participant either fails to do a push-up in the angle required on two non-consecutive repetitions (warning verbalised by an assessor, repetitions counted), fails to maintain movement in time with the metronome, fails to maintain appropriate technique (back straight) or on the volitional failure of the test. To lower body muscular endurance, the volunteer will sit in a chair (regardless of the height of the participant) with his back against the back of the chair. The students will be asked to go from a sitting to a standing position and back to a sitting position for 30 seconds as many times as possible. The test will be performed twice, with an interval of at least 3 minutes between trials, and the highest repetition recorded as the participant’s final score.

Cardiometabolic health

The cardiometabolic variables that will be analysed are glucose, insulin, triglyceride (TG), total cholesterol (TC) and glycated hemoglobin (HbA1c). Blood samples will be collected from the antecubital vein in vacuum tubes after 12 hours fasting, at two different moments (baseline and postintervention), followed the recommendations of the Brazilian Society of Clinical Pathology/Laboratory Medicine. Fasting glucose will be measured using the reference enzyme with Hexokinase. To determine the fasting insulin will be used by the chemiluminescence method. The total cholesterol (TC) and triglycerides (TG) will be analysed by enzymatic colorimetric method, and glycated hemoglobin will be determined using high-performance liquid chromatography. The homeostasis model assessment for insulin resistance (HOMA-IR) will be used to determine insulin sensitivity, and will be calculated using the following formula: [(glucose* 0,0556)*insulin]/22,5([50](#_ENREF_50)).

Cardiorespiratory fitness

CRF will be assessed using PACER FITNESSGRAM test and will be administered following standardized procedures ([51](#_ENREF_51)), which have excellent validity and reliability in this population([52](#_ENREF_52)). Test administrators will provide verbal encouragement during the shuttle to maximise participant motivation. A 20m course will be set up on a hard surface with participants instructed to run back and forth between two sets of lines while keeping pace with a pre-recorded cadence (indicated by a single beep for each 20m shuttle). The test begins at 8.5km/h (slow pace), and increases by 0.5km/hour with each passing minute. The test will be terminated when the participants fail to complete two consecutive laps in the allotted time or voluntarily dropped out due to fatigue. The last successful stage will be recorded and converted into the number of 20m laps completed, and the total number of laps will be used to estimate maximal aerobic capacity (VO2 max) using the equation: 45.619+(0.353*PACER laps)–(1.121*age)([53](#_ENREF_53)).

Mental Health

Psychological difficulties

The ‘Strengths and Difficulties Questionnaire’ (SDQ)([54](#_ENREF_54), [55](#_ENREF_55)) will be used to measure symptoms of mental health disorder. The questionnaire has 25 items which comprise five domains, covering 2 subscales (ie, difficulties and strengths). The difficulties subscale consists of four domains: (i) emotional symptoms (anxiety and depressive symptoms); (ii) conduct problems; (iii) hyperactivity/inattention; (iv) peer relationship problems; and the strengths subscale consists of one domain (v) pro-social behaviour (positive behaviors such as being kind and helpful, scored in reverse of the other subscale). For each item, participants respond using a 3-point scale (ie, ‘not true’=0, ‘somewhat true’=1 and ‘certainly true’=2) and each of the five subscales the score can range from 0 to 10. Lower scores indicate fewer psychological difficulties.

Well-being

The well-being will be measured by two domains of the KIDSCREEN-27 questionnaire, physical well-being consist 5-item and psychological with 7-item. For each item, participants respond using a scale with scores from one to five points([56](#_ENREF_56)) .

Sleep

Sleep quality and quantity will be collected by Pittsburgh Sleep Quality Index (PSQI)([57](#_ENREF_57)), which is validated for Brazilian adolescents([58](#_ENREF_58)). The PSQI is a self-reported questionnaire that asks respondents to report on their sleep quality and signs of sleep disturbance for the 1-month period prior to completing the questionnaire. The PSQI includes 19 questions, categorized into seven groups (sleep quality, sleep latency, sleep duration, habitual sleep efficiency, sleep disturbance, use of sleeping medications and daytime dysfunction). Each constituent question produces a score on a 4-point Likert-type scale (from 0 to 3) and the total score is made up of scores from each of the seven subgroups of questions, giving a cumulated score between 0 and 21.

Anthropometric assessments

Body weight will be measured to the nearest 0.1 kg in light clothing without shoes using a portable digital scale (Welmy®, Santa Bárbara do Oeste, São Paulo, Brazil) and height will record using a portable stadiometer (Welmy®, Santa Bárbara do Oeste, São Paulo, Brazil). Both weight and height will be measured twice to reduce the risk of measurement error. A third measurement will occur it there is there be a difference of >0.1 kg for weight and >0.3 cm for height between the first and second measurement. Body mass index (BMI) will be calculated using the standard equation (weight [kg]/height [m]2) and BMI-z scores will be determine using the ‘LMS’ method according to World Health Organization data ([59](#_ENREF_59)). Waist circumference will be measured at the midpoint between the last rib and the iliac crest using a steel tape (Sanny®, São Bernardo do Campo, São Paulo, Brazil).

**Hypothesised mediators**

Resistance training self-efficacy

RT self-efficacy will be assessed by a four-item scale developed specifically for use with adolescents ([60](#_ENREF_60)), and the participants will respond about their confidence to engage in resistance training using a 5-points Likert (1 = strongly disagree to 5 = strongly agree).

Basic psychological needs satisfaction

The ‘Adolescent Psychological Need Support in Exercise Questionnaire’ will be used to evaluate friends´ and teachers support for exercise([61](#_ENREF_61)). The evaluation of this instrument requires satisfaction during exercise across the three-item: autonomy support, relatedness support and, competence support. Participants reported their satisfaction using a 7-point Likert scale ranging from *1 ‘strongly disagree’ to 7 ‘strongly agree’*.

Autonomous motivation

The 'Behavioural Regulations in Exercise Questionnaire'([62](#_ENREF_62)) will be used to assess autonomous motivation for physical activity using 2-subscales: identified and intrinsic regulations. Adolescents respond on a five-point scale ranging *from 0 ‘not true for me’ to 4 ‘very true for me’*.

**Control Variables**

Somatic maturation

The maturation will be estimated for each participant by predicting years from the attainment of peak height velocity via sex-specific multivariable equations that include stature, sitting height, leg length, body mass, chronological age and their interactions([63](#_ENREF_63)). Leg length will be calculated subtracting sitting height from stature.

**Process evaluation**

The 5 domains will be used to assess feasibility based on the following: 1) consent rate (how many participants offered the program agreed to be involved); 2) retention rate (retention rate at 16 – wk follow-p); 3) attendance (student participation in the structured physical activity), 4) students´ satisfaction with the program (“I enjoyed participating in the RT sessions on 5-point Likert scale: 5=strongly agree to 1=strongly disagree) and 5) engagement ( student engagement with the pedometer self-monitoring and adherence to goals setting).

**STATISTICAL ANALYSES**

Linear mixed models will be used to analyse the primary and secondary outcomes using IBM SPSS Statistics for Windows (version 20.0; 2010 SPSS Inc, IBM Company, Armonk, NY 2010 SPSS Inc., IBM Company, Armonk, NY), with significance set at P< 0.05. The models will be used to assess the effect of treatment (or IG or control), time (baseline, 4 months and 12-month) and the group-by-time interaction, weighted for sex and adjusted for the covariates maturation and BMI. Mixed model analyses are consistent with the intention-to-treat principle, assuming the data are missing at random. Effect sizes between groups will be calculated using Cohen's *d* (the adjusted difference between the control and intervention groups over time divided by the pooled standard deviation of change) and interpreted as follows: *d* = 0.2 (small), *d* = 0.5 (medium), and *d* = 0.8(large). Potential moderators will be explored using linear mixed models with interaction terms for the following: sex (male, female), socioeconomic status (low, medium, high), initial weight status (healthy weight vs overweight/obese). Subgroup analyses will be only conducted if significant interaction effects P≤0.10. Hypothesised mediators of physical activity behaviour change will be examined using multilevel linear analysis.

**POSSIBLE RESULTS**

Considering the several benefits promoted by the regular practice of physical activity reported in the literature, it is expected that the PA promotion program will increase the levels of physical activity in adolescents and, consequently, promote improvements in indicators of physical fitness, cardiometabolic and mental health.

**REFERENCE:**

1. Hallal PC, Victora CG, Azevedo MR, Wells JC. Adolescent physical activity and health: a systematic review. Sports medicine (Auckland, NZ). 2006;36(12):1019-30.

2. WHO. WHO Guidelines Approved by the Guidelines Review Committee. Global Recommendations on Physical Activity for Health. Geneva: World Health Organization

Copyright (c) World Health Organization 2010.; 2010.

3. Rhodes RE, Janssen I, Bredin SSD, Warburton DER, Bauman A. Physical activity: Health impact, prevalence, correlates and interventions. Psychology & Health. 2017;32(8):942-75.

4. Werneck AO, Oyeyemi AL, Fernandes RA, Romanzini M, Ronque ERV, Cyrino ES, et al. Regional Socioeconomic Inequalities in Physical Activity and Sedentary Behavior Among Brazilian Adolescents. Journal of physical activity & health. 2018;15(5):338-44.

5. Silva PRd, Santos GCd, Faria WFd, Corrêa RC, Elias RGM, Stabelini Neto A. Tracking of physical activity in adolescents between 2010 and 2014. Revista Brasileira de Cineantropometria & Desempenho Humano. 2018;20(1):64-70.

6. Dumith SC, Gigante DP, Domingues MR, Kohl HW, III. Physical activity change during adolescence: a systematic review and a pooled analysis. International Journal of Epidemiology. 2011;40(3):685-98.

7. Boddy LM, Thomas NE, Fairclough SJ, Tolfrey K, Brophy S, Rees A, et al. ROC Generated Thresholds for Field-Assessed Aerobic Fitness Related to Body Size and Cardiometabolic Risk in Schoolchildren. PLOS ONE. 2012;7(9):e45755.

8. Ross R, Blair SN, Arena R, Church TS, Despres JP, Franklin BA, et al. Importance of Assessing Cardiorespiratory Fitness in Clinical Practice: A Case for Fitness as a Clinical Vital Sign: A Scientific Statement From the American Heart Association. Circulation. 2016;134(24):e653-e99.

9. Smith JJ, Eather N, Morgan PJ, Plotnikoff RC, Faigenbaum AD, Lubans DR. The health benefits of muscular fitness for children and adolescents: a systematic review and meta-analysis. Sports medicine (Auckland, NZ). 2014;44(9):1209-23.

10. Ortega FB, Ruiz JR, Castillo MJ, Sjostrom M. Physical fitness in childhood and adolescence: a powerful marker of health. International journal of obesity (2005). 2008;32(1):1-11.

11. Collings PJ, Westgate K, Vaisto J, Wijndaele K, Atkin AJ, Haapala EA, et al. Cross-Sectional Associations of Objectively-Measured Physical Activity and Sedentary Time with Body Composition and Cardiorespiratory Fitness in Mid-Childhood: The PANIC Study. Sports medicine (Auckland, NZ). 2017;47(4):769-80.

12. Marques A, Santos R, Ekelund U, Sardinha LB. Association between physical activity, sedentary time, and healthy fitness in youth. Medicine and science in sports and exercise. 2015;47(3):575-80.

13. Prevention. CfDCa. Comprehensive school physical activity programs: a guide for schools. In: Services UDoHaH, editor. Atlanta, GA2013.

14. Tremblay MS, Barnes JD, Gonzalez SA, Katzmarzyk PT, Onywera VO, Reilly JJ, et al. Global Matrix 2.0: Report Card Grades on the Physical Activity of Children and Youth Comparing 38 Countries. Journal of physical activity & health. 2016;13(11 Suppl 2):S343-s66.

15. Silva DAS, Chaput JP, Tremblay MS. Participation frequency in physical education classes and physical activity and sitting time in Brazilian adolescents. PLoS One. 2019;14(3):e0213785.

16. Telford RD, Cunningham RB, Fitzgerald R, Olive LS, Prosser L, Jiang X, et al. Physical education, obesity, and academic achievement: a 2-year longitudinal investigation of Australian elementary school children. American journal of public health. 2012;102(2):368-74.

17. Standal ØF, Aggerholm K. Habits, skills and embodied experiences: a contribution to philosophy of physical education. Sport, Ethics and Philosophy. 2016;10(3):269-82.

18. Hollis JL, Williams AJ, Sutherland R, Campbell E, Nathan N, Wolfenden L, et al. A systematic review and meta-analysis of moderate-to-vigorous physical activity levels in elementary school physical education lessons. Preventive Medicine. 2016;86:34-54.

19. da Costa BGG, da Silva KS, da Silva JA, Minatto G, de Lima LRA, Petroski EL. Sociodemographic, biological, and psychosocial correlates of light- and moderate-to-vigorous-intensity physical activity during school time, recesses, and physical education classes. Journal of sport and health science. 2019;8(2):177-82.

20. Buchan DS, Ollis S, Thomas NE, Buchanan N, Cooper S-M, Malina RM, et al. Physical activity interventions: effects of duration and intensity. Scandinavian journal of medicine & science in sports. 2011;21(6):e341-e50.

21. Love R, Adams J, van Sluijs EMF. Are school-based physical activity interventions effective and equitable? A meta-analysis of cluster randomized controlled trials with accelerometer-assessed activity. Obesity Reviews. 2019;20(6):859-70.

22. Dobbins M, Husson H, DeCorby K, LaRocca RL. School-based physical activity programs for promoting physical activity and fitness in children and adolescents aged 6 to 18. The Cochrane database of systematic reviews. 2013(2):Cd007651.

23. Bauman AE, Reis RS, Sallis JF, Wells JC, Loos RJF, Martin BW, et al. Correlates of physical activity: why are some people physically active and others not? Lancet. 2012;380(9838):258-71.

24. Martins J, Marques A, Sarmento H, Carreiro da Costa F. Adolescents' perspectives on the barriers and facilitators of physical activity: a systematic review of qualitative studies. Health Educ Res. 2015;30(5):742-55.

25. Corder K, Atkin AJ, Ekelund U, van Sluijs EM. What do adolescents want in order to become more active? BMC public health. 2013;13:718.

26. Hulteen RM, Smith JJ, Morgan PJ, Barnett LM, Hallal PC, Colyvas K, et al. Global participation in sport and leisure-time physical activities: A systematic review and meta-analysis. Prev Med. 2017;95:14-25.

27. Lubans DR, Morgan PJ, Okely AD, Dewar D, Collins CE, Batterham M, et al. Preventing Obesity Among Adolescent Girls: One-Year Outcomes of the Nutrition and Enjoyable Activity for Teen Girls (NEAT Girls) Cluster Randomized Controlled Trial. Archives of pediatrics & adolescent medicine. 2012;166(9):821-7.

28. Dewar DL, Morgan PJ, Plotnikoff RC, Okely AD, Collins CE, Batterham M, et al. The nutrition and enjoyable activity for teen girls study: a cluster randomized controlled trial. American journal of preventive medicine. 2013;45(3):313-7.

29. Smith JJ, Morgan PJ, Plotnikoff RC, Dally KA, Salmon J, Okely AD, et al. Smart-phone obesity prevention trial for adolescent boys in low-income communities: the ATLAS RCT. Pediatrics. 2014;134(3):e723-31.

30. Kennedy SG, Smith JJ, Morgan PJ, Peralta LR, Hilland TA, Eather N, et al. Implementing Resistance Training in Secondary Schools: A Cluster Randomized Controlled Trial. Medicine and science in sports and exercise. 2018;50(1):62-72.

31. Lubans DR, Smith JJ, Morgan PJ, Beauchamp MR, Miller A, Lonsdale C, et al. Mediators of Psychological Well-being in Adolescent Boys. The Journal of adolescent health : official publication of the Society for Adolescent Medicine. 2016;58(2):230-6.

32. Filho VCB, Silva KSd, Mota J, Beck C, Lopes AdS. A Physical Activity Intervention for Brazilian Students From Low Human Development Index Areas: A Cluster-Randomized Controlled Trial. 2016;13(11):1174.

33. Costa B, Silva KSD, Silveira PMD, Berria J, Machado AR, Petroski EL. The effect of an intervention on physical activity of moderate-and-vigorous intensity, and sedentary behavior during adolescents' time at school. Brazilian journal of epidemiology

2019;22:e190065.

34. Leme AC, Lubans DR, Guerra PH, Dewar D, Toassa EC, Philippi ST. Preventing obesity among Brazilian adolescent girls: Six-month outcomes of the Healthy Habits, Healthy Girls-Brazil school-based randomized controlled trial. Prev Med. 2016;86:77-83.

35. Moher D, Hopewell S, Schulz KF, Montori V, Gotzsche PC, Devereaux PJ, et al. CONSORT 2010 explanation and elaboration: updated guidelines for reporting parallel group randomised trials. BMJ (Clinical research ed). 2010;340:c869.

36. Corder K, Brown HE, Schiff A, van Sluijs EMF. Feasibility study and pilot cluster-randomised controlled trial of the GoActive intervention aiming to promote physical activity among adolescents: outcomes and lessons learnt. BMJ open. 2016;6(11):e012335.

37. Corder K, Sharp SJ, Atkin AJ, Griffin SJ, Jones AP, Ekelund U, et al. Change in objectively measured physical activity during the transition to adolescence. Br J Sports Med. 2015;49(11):730-6.

38. ABEP. Associação Brasileira de Empresas de Pesquisa - Critério Brasil: Estrato Sócio Econômico

2019.

39. Lubans DR, Smith JJ, Peralta LR, Plotnikoff RC, Okely AD, Salmon J, et al. A school-based intervention incorporating smartphone technology to improve health-related fitness among adolescents: rationale and study protocol for the NEAT and ATLAS 2.0 cluster randomised controlled trial and dissemination study. BMJ open. 2016;6(6):e010448.

40. Bandura A. Health promotion by social cognitive means. Health education & behavior : the official publication of the Society for Public Health Education. 2004;31(2):143-64.

41. Deci EL, Ryan RM. The "What" and "Why" of Goal Pursuits: Human Needs and the Self-Determination of Behavior. Psychological Inquiry. 2000;11(4):227-68.

42. Morgan PJ, Young MD, Smith JJ, Lubans DR. Targeted Health Behavior Interventions Promoting Physical Activity: A Conceptual Model. Exercise and sport sciences reviews. 2016;44(2):71-80.

43. Lubans DR, Lonsdale C, Cohen K, Eather N, Beauchamp MR, Morgan PJ, et al. Framework for the design and delivery of organized physical activity sessions for children and adolescents: rationale and description of the 'SAAFE' teaching principles. The international journal of behavioral nutrition and physical activity. 2017;14(1):24.

44. Kantanista A, Bronikowski M, Laudanska-Krzeminska I, Krol-Zielinska M, Osinski W. Positive effect of pedometer-based walking intervention on body image and physical activity enjoyment in adolescent girls. Biomedical Human Kinetics. 2017;9(1):34-42.

45. Service HP. Parent Guide: how you can support physical activity and weelbeing for your tennegers. In: Health Promotion Service- Directorate of Planning PHaE, editor. South Eastern Sydney Local Health District, Australia2016.

46. Evenson KR, Catellier DJ, Gill K, Ondrak KS, McMurray RG. Calibration of two objective measures of physical activity for children. Journal of sports sciences. 2008;26(14):1557-65.

47. Masse LC, Fuemmeler BF, Anderson CB, Matthews CE, Trost SG, Catellier DJ, et al. Accelerometer data reduction: a comparison of four reduction algorithms on select outcome variables. Medicine and science in sports and exercise. 2005;37(11 Suppl):S544-54.

48. Cooper. Fitnessgram: Test administration manual. Champaign, IL: Human Kinetics1999.

49. Bohannon RW. Sit-to-stand test for measuring performance of lower extremity muscles. Perceptual and motor skills. 1995;80(1):163-6.

50. Oliveira EPd, Souza MLAd, Lima MdDAd. Índice HOMA (homeostasis model assessment) na prática clínica: uma revisão. Jornal Brasileiro de Patologia e Medicina Laboratorial. 2005;41:237-43.

51. Meredith MD, GJ W. FitnessGram & ActivityGram test administration manual. Champaign: IL: Human Kinetics. 2010;Update 4 th ed.

52. Lang JJ, Tomkinson GR, Janssen I, Ruiz JR, Ortega FB, Leger L, et al. Making a Case for Cardiorespiratory Fitness Surveillance Among Children and Youth. Exercise and sport sciences reviews. 2018;46(2):66-75.

53. Mahar MT, Welk GJ, Rowe DA. Estimation of aerobic fitness from PACER performance with and without body mass index. Measurement in Physical Education and Exercise Science. 2018;22(3):239-49.

54. Fleitlich-Bilyk B, Goodman R. Prevalence of Child and Adolescent Psychiatric Disorders in Southeast Brazil. Journal of the American Academy of Child & Adolescent Psychiatry. 2004;43(6):727-34.

55. Goodman R. Psychometric properties of the strengths and difficulties questionnaire. Journal of the American Academy of Child and Adolescent Psychiatry. 2001;40(11):1337-45.

56. Farias Júnior JCd, Loch MR, Lima Neto AJd, Sales JM, Ferreira FELdL. Reprodutibilidade, consistência interna e validade de construto do KIDSCREEN-27 em adolescentes brasileiros. Cadernos de Saúde Pública. 2017;33:e00131116.

57. Buysse DJ, Reynolds CF, 3rd, Monk TH, Berman SR, Kupfer DJ. The Pittsburgh Sleep Quality Index: a new instrument for psychiatric practice and research. Psychiatry research. 1989;28(2):193-213.

58. Passos MH, Silva HA, Pitangui AC, Oliveira VM, Lima AS, Araújo RC. Reliability and validity of the Brazilian version of the Pittsburgh Sleep Quality Index in adolescents. J Pediatr (Rio J). 2017;93(2):200-6.

59. WHO. WHO Multicentre Growth Reference Study Group. WHO Growth reference data 5-19 years: BMI-for-age (5-19 years). Geneva: World Health Organization. 2007.

60. Lubans DR, Morgan P, Callister R, Plotnikoff RC, Eather N, Riley N, et al. Test-retest reliability of a battery of field-based health-related fitness measures for adolescents. Journal of sports sciences. 2011;29(7):685-93.

61. Emm-Collison LG, Standage M, Gillison FB. Development and Validation of the Adolescent Psychological Need Support in Exercise Questionnaire. Journal of sport & exercise psychology. 2016;38(5):505-20.

62. Markland D, Tobin V. A modification to the Behavioural Regulation in Exercise Questionnaire to include an assessment of amotivation. Journal of sport & exercise psychology. 2004;26(2):191-6.

63. Mirwald RL, Baxter-Jones AD, Bailey DA, Beunen GP. An assessment of maturity from anthropometric measurements. Medicine and science in sports and exercise. 2002;34(4):689-94.

**DOCUMENTS OF CONSENT FOR PARTICIPATION IN A RESEARCH PROJECT**

Annex I: **AUTHORIZATION OF THE FAMILY OR GUARDIAN** – CONSENT FORM

Dear Mr./Mrs.,

a) Your child is being invited to participate in a study entitled “**Effects of a physical activity promotion program on the level of physical activity, physical fitness, cardiometabolic and mental health in adolescents**”.

b) The aim of this study is to evaluate the effects of a physical activity promotion program on the level of physical activity, muscles, physical conditioning, glucose (blood sugar) and lipids (blood fat), and mental ( signs and symptoms of anxiety, stress, depression, peer relationships) in adolescents.

c) The participation of your child (a) is very important, as he/she meets the study's inclusion criteria, which include: enrolled in the 8th or 9th grade of elementary school II; age between 13 and 14 years; have informed consent form signed by the responsible and by the own participant. As soon as you authorize your child's participation, he/she will carry out evaluations as follows: respond to an anamnesis (name; date of birth; name of guardians); physical activity questionnaire. Afterwards, you will do to measurements of your weight, height, waist and hip circumferences, motor tests and blood tests at the beginning of the research and after 4 months and one year after the end of the intervention. Regarding blood collection, it will be carried out by a registered nurse and later sent to the contracted laboratory for clinical analysis in Jacarezinho-Pr, the blood samples will be get out soon after the blood analysis is performed. Right after the initial assessment, participants will be randomly distributed (draw) into an experimental or control group. Participants in the experimental group will receive an intervention program immediately after the initial assessment, while participants in the control group will receive the intervention program immediately after the follow-up assessment.

d) The intervention program (that your child will participate) will have a duration of 4 months, comprising a session of structured physical activity, self-monitoring and guidance on a healthy lifestyle. The structured physical activity session will take place during school physical education classes under the supervision of auxiliary researchers, which will include exercises to improve muscle strength/endurance and cardiorespiratory conditioning. Regarding self-monitoring, the participant will receive an instrument (device) called a pedometer, which aims to mark the number of steps per day performed by the individual, helping in self-monitoring to acquire an active lifestyle. This appliance should be worn at hip height (at the waistband of pants/shorts) and should be worn daily throughout the day, except for sleeping, swimming and take a shower. When withdrawing, the adolescent must record the number of steps accumulated during the day in a diary that will also be delivered with the device. Guidance on a healthy lifestyle, messages will be sent through the WhatsApp® application to encourage healthy behavior. All participants will do in the same evaluations carried out at the beginning of the project, after the 4-month intervention and one year of follow-up.

e) All the information you want is assured, before, during and after the study. Besides, participant will have access to the results of laboratory tests and the final result of the research, if desired.

f) If your child does not achieve the expected results, we will forward it to the Family Health Strategy (FHS).

g) Your child's participation (a) is voluntary. You are free to refuse to participate in the study, or withdraw your consent at any time. In addition, the participant has the right to withdraw from the research at any time, without causing any harm.

h) All expenses necessary to carry out the research are not the responsibility of the participant or his/her guardian, but of the responsible researchers. If the participant has any expense related to participation in the research, he will be entitled to reimbursement, paid by the research team.

i) Adolescents will be informed about the risks of sedentary behavior. The expected benefits of the intervention are behavior change in lifestyle, which include: Increased physical activity level in adolescents; lowering blood sugar and fat levels; improvement in musculature and physical conditioning (cardiorespiratory), decrease in sedentary behavior; eating habits, psychological control (self-control; self-motivation; interpersonal relationships).

j) This intervention does not anticipate risks and injuries for participating in the study, where the risks are trivial. Regarding the evaluation, all questionnaires will be applied in a self-reported way, that is, the participant will answer individually and according to their own interpretation. Thus, the adolescent will NOT be exposed or compared to their peers, avoiding any kind of embarrassment and psychological disorders during the assessment. Muscle pain due to physical tests, all tests will be conducted and supervised by experienced researchers who will demonstrate to the participants how the movements should be performed, thus minimizing any type of injury due to the execution of wrong movement. As for blood collection, the risks are also minimal (minor pain at the time of blood pulsation), as the collection will be carried out in an appropriate place, by specialized people (nurses), but in case of any incident, the adolescent together with his guardian (who must be present on the day of collection) will be taken immediately to Santa Casa de Misericórdia in Jacarezinho. Regarding the intervention, possible muscle pain may occur due to the exercises inserted during the physical education class and the increase in the number of steps (physical activity intervention). However, to alleviate and/or minimize this discomfort, muscle warm-up will be performed before starting the exercise and stretching at the end of each session; muscle rest time (48 hours after activity) will also be respected. In addition, teenagers will be advised when to adopt a healthy diet and daily hydration.

h) When the results are published, the name of the participants will not appear, but a code.

i) The participant has a right compensation for damages arising from the research, under the terms of the law.

j) The consent form will be written in two copies, initialed on all pages and signed by the researcher, one copy for the researcher and the other for the participant.

If you have questions or need more information about the survey, you can contact us: Prof. Dr. **Antonio Stabelini Neto**. **Center for Health Sciences of the State University of Northern Paraná.** Alameda Padre Magno, 841 - Jacarezinho - PR - CEP 86400-000. Tel.: (043) 3525-0498. E-mail: asneto@uenp.edu.br. Prof. Mother Géssika Castilho dos Santos. Alameda Padre Magno, 841 - Jacarezinho - PR - CEP 86400-000. Tel.: (043) 3525-0498 or (43) 99900-0265. E-mail: gessika.castilho@gmail.com.

If you have any questions or complaints of an ethical nature, you can contact the Ethics Committee (CEP/UENP, Rod. BR 369, Km 54 - Bandeirantes-PR, CEP 86360-000, Caixa Postal 261, Phone (43)3542-8056), Open from Monday to Friday from 7:30 am to 12:00 pm and from 1:30 pm to 5:00 pm. This term must be completed in two copies of equal content, one of which is duly completed, signed and delivered to the volunteer.

I,____________________________________________ have read and agree that my child____________________________________________________ participate in the survey.

____________________________________________

Parents or guardian signature or fingerprint

Date: ___/___/____

I, Antonio Stabelini Neto, declare that I provided all the information regarding the research project.

____________________________________________

Antonio Stabelini Neto

Date: ___/___/____

I, Géssika Castilho dos Santos, declare that I have provided all the information regarding the research project.

____________________________________________

Géssika Castilho dos SANTOS

Date: ___/___/____

Jacarezinho, ___ of ________ of 202_.

Annex II: **AUTHORIZATION OF ADOLESCENTS-** TERM OF ASSENT

What does assent mean?

Assent means that you agree to take part in a group of adolescents, of your own age, to participate in a survey. Your rights will be respected and you will receive all the information as simple as it may seem. It may be that this document called TERM OF ASSENT contains words that you do not understand. Please ask the research officer or study staff to explain any words or information that you do not clearly understand.

Dear adolescents:

You have been invited to participate in the survey “Effects of a physical activity promotion program on the level of physical activity, physical fitness, cardiometabolic and mental health in adolescents”. Your parents have allowed you to participate.

We want to know if the physical activity program will promote an improvement in muscle strength/endurance and fitness, improve blood sugar and fat levels, improve levels of anxiety, stress, social interaction and increase physical activity, and teenagers who will participate in this research are between 13 and 14 years of age. You don't need to participate in the survey if you don't want to, it's your right, you won't have any problem if you give up. The survey will be conducted at the school during physical education classes, where participants will participate as follows:

- First the participant:

1) Will respond to a questionnaire about personal information; socioeconomic status and a questionnaire on the determinants of physical activity. They will undergo physical activity assessments, physical tests, answer questionnaires, measurements of their weight, height, waist circumferences, and blood tests. Blood withdrawal will be performed 3 times at different times by a registered nurse and soon after forwarded to the private laboratory for clinical analysis in Jacarezinho-Pr. Once the blood test is performed, your blood sample will be discarded. The evaluations will take place at 3 different times: beginning and end of the research, and after 1 year.

- After the initial assessment:

2) Participants will be distributed by drawing lots in an experimental or control group, that is, you can be from the experimental or control group. Everyone will receive the intervention. Participants in the experimental group will first participate in the 4-month physical activity intervention program, which consists of a structured physical activity session, use of a device to monitor the number of steps per day, and guidance on a lifestyle active. While the participants who are in the control group will participate in the intervention one year after the end of the intervention in the experimental group.

3) You will participate in a structured session in physical education classes for 4 months, each session lasting 20 minutes. In this physical activity session, you will choose which exercises you want to perform, which include: muscle strengthening (eg squats, push-ups, sit-ups) and cardio-respiratory conditioning (eg jumping jacks, jumping rope). During the entire session you will be supervised by your physical education teacher and an assistant researcher/assistant. You will also receive a device called a pedometer to help you monitor the number of steps you take each day. This appliance should be worn at hip height (at the waistband of pants/shorts/shorts) and worn throughout the day, except for sleeping, swimming and showering. This device should be put on in the morning and removed at bedtime, and when removing the adolescent should note the number of steps accumulated during the day in a diary that will also be delivered with the device. You will also receive weekly messages through the WhatsApp® app about healthy behavior.

- After the intervention:

4) You will perform again the same assessments done at the beginning of the project.

5) If you do not achieve the expected result (improvement in muscle strength/endurance; improvement in physical conditioning; improvement in blood sugar and fat levels; improvement in social interaction), we will forward you to the Family Health Strategy ( FHS).

- IT IS IMPORTANT for you to know that:

6) You will be entitled to compensation for any damages arising from the research, under the terms of the law.

7) You will be informed and will have access to the results of laboratory tests and the final result of the research.

We make it clear that no one will know that you are taking part in the survey, we will not tell others, or give outsiders the information you give us. The survey results will be published, but without identifying the teenagers who participated in the survey.

We further clarify that you will not pay or be remunerated for your participation. In addition, it is noteworthy that if there are expenses, you will be reimbursed by the

researcher.

The physical activity assessment and program are considered safe, but minimal “risks” are possible. Regarding the evaluation, all questionnaires will be applied in a self-reported way, that is, the participant will answer individually and according to his/her own interpretation. Thus, the adolescent will NOT be exposed or compared to their peers, avoiding any kind of embarrassment and psychological disorders during the assessment. Muscle pain due to physical tests, all tests will be conducted and supervised by experienced researchers who will demonstrate to the participants how the movements should be performed, thus minimizing any type of injury due to the execution of wrong movement. As for blood collection, the risks are also minimal (mild pain at the time of blood pulsation), as the collection will be carried out in an appropriate place, by specialized people (nurses), but in case of any incident, the adolescent together with his guardian (who must be present on the day of collection) will be taken immediately to Santa Casa de Misericórdia in Jacarezinho. Regarding the intervention, possible muscle pain may occur due to the exercises inserted during the physical education class and the increase in the number of steps (physical activity intervention). However, to alleviate and/or minimize this discomfort, muscle warm-up will be performed before starting the exercise and stretching at the end of each session; muscle rest time (48 hours after activity) will also be respected. In addition, teenagers will be advised when to adopt a healthy diet and daily hydration. If something goes wrong during the intervention, you can contact us by phone ((043) 3525-0498 or 99900-0265) of the researcher (Géssika Castilho dos Santos). As for blood collection, the risks are also minimal (mild pain at the time of blood pulsation), as the collection will be carried out in an appropriate place, by specialized people (nurses), but in case of any incident, you and your guardian (who must be present on the day of collection) they will be taken immediately to Santa Casa de Misericórdia in Jacarezinho.

But there are good things that can happen such as: without any expense, you will receive information about the risks of sedentary behavior; will participate in exercises that will improve your muscles (muscle fitness) and will use a pedometer to help change lifestyle behaviors, which will result in several benefits: increased physical activity level in adolescents; reduction in blood sugar and fat levels, improvement in eating habits and psychological control (self-control; self-motivation; interpersonal relationships).

If you have questions or need more information about the survey, you can contact us: Prof. Dr. Antonio Stabelini Neto. Center for Health Sciences of the State University of Northern Paraná. Alameda Padre Magno, 841 - Jacarezinho - PR - CEP 86400-000. Tel.: (043) 3525-0498. E-mail: asneto@uenp.edu.br. Prof. Mother Géssika Castilho dos Santos. Alameda Padre Magno, 841 - Jacarezinho - PR - CEP 86400-000. Tel.: (043) 3525-0498 or (43) 99900-0265. E-mail: gessika.castilho@gmail.com.

If you have any questions or complaints of an ethical nature, you can contact the Ethics Committee (CEP/UENP, Rod. BR 369, Km 54 - Bandeirantes-PR, CEP 86360-000, Caixa Postal 261, Phone (43)3542-8056), Open from Monday to Friday from 7:30 am to 12:00 pm and from 1:30 pm to 5:00 pm.

This term must be completed in two copies of equal content, one of which is duly completed, signed and delivered to the volunteer.

I ___________________________________ I have read and agree to participate in the study as a volunteer.

_________________________________

Signature of participating adolescent or fingerprint

Date: ___/___/____

I, Antonio Stabelini Neto, declare that I provided all the information regarding the research project.

____________________________________________

Antonio Stabelini Neto

Date: ___/___/____

I, Géssika Castilho dos Santos, declare that I have provided all the information regarding the research project.

____________________________________________

Géssika Castilho dos SANTOS

Date: ___/___/____

Jacarezinho, ___ of ________ of 202_.
