# Supplementary material for: Improving physical activity behaviors, physical fitness, cardiometabolic and mental health in adolescents - ActTeens Program: A protocol for a randomized controlled trial
Source: PLoS One. 2022 Aug 9;17(8):e0272629. doi: 10.1371/journal.pone.0272629 (PMC9362910; doi:10.1371/journal.pone.0272629)
Supplement: S2 File — (DOCX) [file pone.0272629.s003.docx]

**Efeitos de um programa de promoção da atividade física no nível de atividade física, aptidão física, saúde cardiometabólica e mental em adolescentes**

**Introdução**

A prática regular de atividade física (AF) tem sido associada com inúmeros benefícios à saúde, os quais incluem: a melhora da aptidão cardiorrespiratória (APC), composição corporal, perfil metabólico e saúde mental ([1](#_ENREF_1), [2](#_ENREF_2)). Recomendações globais sobre atividade AF sugere que crianças e adolescentes devem realizar no mínimo 60 minutos de AF moderada a vigorosa (AFMV) por dia([2](#_ENREF_2)),o qual deve incluir atividades de fortalecimento muscular (ex: treinamento de resistência) em pelo menos 3 vezes na semana. Entretanto, estudos têm apontado que 81% dos adolescentes de idade escolar (11 – 17 anos) não cumprem estas recomendações([3](#_ENREF_3)), e no Brasil, apenas 8.4% dos adolescentes são considerados fisicamente ativos ([4](#_ENREF_4)). Além disso, estudos longitudinais([5-7](#_ENREF_5)) vêm observando um declínio do nível da atividade física e dos componentes da aptidão física relacionada à saúde (cardiorrespiratória e muscular) durante a adolescência, resultando em uma grande preocupação, pois ambos são considerados importantes preditores relacionado à saúde.

Evidências prévias([8](#_ENREF_8), [9](#_ENREF_9)) observaram que baixos níveis de aptidão cardiorrespiratória e força muscular são relacionados com fatores de riscos para doenças crônicas. É importante ressaltar que a aptidão física (APF) na infância e adolescência têm sido considerada como um potente preditor para a saúde na vida adulta([10](#_ENREF_10)), desta forma, é essencial a manutenção da APF dentro do nível saudável, o qual é obtido por meio da atividade física (AF). Resultados consistentes mostraram uma associação entre altos níveis de AFMV e bons índices cardiorrespiratório e força muscular em adolescentes ([11](#_ENREF_11), [12](#_ENREF_12)). Portanto, intervenções objetivando a promoção da atividade e a melhora da aptidão física em adolescentes devem ser a prioridade de saúde pública.

Ambiente escolar é o lugar onde os adolescentes passam cerca de cinco horas por dia adquirindo conhecimentos sobre diferentes tópicos, o qual também inclui o tema saúde. Nesta perspectiva, as escolas são consideradas um local ideal para promover informar aos estudantes sobre os benefícios da prática regular de atividade física ([13](#_ENREF_13), [14](#_ENREF_14)), além de ter o papel fundamental de criar oportunidades de atividades físicas estruturadas e não estruturadas, e assim, promover e estimular a mudança de comportamento ([14](#_ENREF_14)). Uma oportunidade para isto acontecer é através das aulas educação física (ED.F) que representa um meio ideal tanto para o ensino como para a prática, o qual auxilia na aquisição e consolidação de um hábitos ativo que poderá ser transferido para a vida adulta ([13](#_ENREF_13)). Além disso, esta oportunidade no ambiente escolar poderá proporciona aos adolescentes uma vivência em relação prática de AF, a qual eles podem não ter fora do contexto escolar.

Estudos têm mostrado que adolescentes com alta frequência de participação em aulas de ED.F tem gasto mais tempo em AFMV e menos tempo sentado durante o dia ([15](#_ENREF_15)), melhor desempenho acadêmico([16](#_ENREF_16)), melhor interação social e baixo risco cardiometabólico ([17](#_ENREF_17)) quando comparado com seus pares que não realizam as aulas de educação física. Entretanto, uma revisão sistemática e meta-análise([18](#_ENREF_18)) investigou o tempo gasto em AFMV durante a educação física escolar, os resultados verificaram que apenas 40.5% do tempo das aulas é gasto em AFMV pelos estudantes. Em relação aos adolescentes brasileiros, essa proporção de tempo em AFMV é ainda menor, cerca de 16%([19](#_ENREF_19)) . Uma possível explicação para essa observação pode ser o fato das aulas de educação física ter foco somente sobre modalidades esportivas e jogos tradicionais que visam a competitividade([20](#_ENREF_20)), a qual pode não ser um caminho efetivo e agradável para alguns adolescentes se engajar na prática atividade física.

Apesar do potencial que a escola tem para influenciar um comportamento saudável, intervenções prévias de base escolar direcionadas à adolescentes obtiveram resultados mistos([21](#_ENREF_21), [22](#_ENREF_22)), uma possível justificativa para essa inconstância nos achados pode ser atribuído ao fato dos pesquisadores não considerar fatores chaves na elaboração da intervenção, tais como facilitadores e barreiras para a participação na atividade física ([23](#_ENREF_23)). Diante disso, para desenvolver uma intervenção efetiva é necessário considerar fatores que influencie a atividade física dos adolescentes como prazer, competência percebida, motivação, suporte social (professores, amigos e pais), acessibilidade e disponibilidade de novas oportunidades de práticas ([23](#_ENREF_23), [24](#_ENREF_24)).

Entretanto, poucos estudos de intervenção estão abordando novas (diferentes) oportunidades de práticas que não seja as atividades tradicionais. Em adição, dados globais referentes à adolescentes têm enfatizado o desejo e a vontade dos mesmo em experimentar atividades físicas não convencionais como treinamento de resistência e aulas de ginásticas ([25](#_ENREF_25), [26](#_ENREF_26)). É importante destacar que estas atividades são consideradas as mais praticadas com o aumento da idade, nesta perspectiva, a inserção destas atividades no ambiente escolar contribuirá substancialmente para a prática na vida adulta ([26](#_ENREF_26)).

Dada a capacidade das escolas em proporcionar aos estudantes o desenvolvimento de habilidades e confiança necessária para o engajamento em atividades físicas ao longo da vida ([25](#_ENREF_25)) e, considerando a ausência de atividades físicas alternativas nas intervenções/programas escolares, há a necessidade de introduzir atividades comumente praticadas por toda a vida, como por exemplo, treinamento de resistência (TR). Diante desta perspectiva, atividades físicas de condicionamento físico parece ser um mediador adequado para atender a recomendação mínima necessária de AF diária.

No geral, intervenções em atividade física para jovens descritas na literatura têm focado predominantemente no componente aeróbico das diretrizes, sendo que poucas pesquisas têm trabalhado a aptidão muscular (APM). Diante destas limitações, algumas intervenções efetivas ([27-30](#_ENREF_27)) incluindo TR em adolescentes tem sido conduzida no ambiente escolar. Resumidamente, os resultados obtidos destas intervenções foram a redução no tempo de tela ([27](#_ENREF_27), [30](#_ENREF_30)) e melhora na composição corporal ([28](#_ENREF_28)), aptidão muscular ([28-30](#_ENREF_28)), competência das habilidades no treinamento de resistência ([29](#_ENREF_29), [30](#_ENREF_30)), auto-eficácia e motivação autônoma para atividade física ([29](#_ENREF_29)), e bem-estar ([31](#_ENREF_31)). Entretanto, um número limitado de programas de AF no ambiente escolar tem sido desenvolvido em países de baixa-média renda, como o Brasil.

No Brasil, programas de base escolar ([32-34](#_ENREF_32)) tem sido desenvolvido como objetivo sobre a promoção de um comportamento ativo, porém nenhum programa teve como enfoque a aptidão muscular. Desta forma, considerando que (i) adolescência é um período de transição social e biológico, onde os padrões futuros da saúde adulta são estabelecidos, (ii) os benefícios da AF e o seu declínio entre os adolescentes, (iii) o importante papel da aptidão muscular para a saúde, e (iv) escola é um ambiente ideal para promover novas oportunidades de práticas de AF, torna-se necessário desenvolver um programa de base escolar com enfoque na aptidão muscular por meio de atividades de fortalecimento muscular.

**Objetivos do estudo**

Objetivo primário:

- Verificar o efeito do programa de atividade física de base escolar sobre a aptidão muscular dos adolescentes.

Secundários:

- Avaliar a viabilidade e a eficácia preliminar do programa sobre a melhora da aptidão física relacionado à saúde, saúde mental e cardiometabólicas, e o aumento no nível de atividade física em escolares.

- Analisar potenciais mediadores da mudança do comportamento da atividade física.

**MÉDOTOS**

**Delineamento do estudo**

Esse projeto tem como característica um delineamento experimental, um ensaio clínico randomizado com duração de 16 semanas, com componentes múltiplos de promoção da atividade física para adolescentes, sendo que o design, a conduta e os relatórios seguirão as diretrizes do CONSORT (Consolidated Standards of Reporting Trials)([35](#_ENREF_35)). O presente estudo iniciará no primeiro semestre de 2022 na cidade de Jacarezinho, Paraná, sendo que a coleta de dados ocorrerá em três momentos distintos: linha de base (fevereiro de 2022) e pós intervenção (Junho de 2022) e follow-up (fevereiro de 2023).

**População , recrutamento e seleção das escolas**

Serão convidados a participar da pesquisa todas as escolas de Jacarezinho com estudantes de 13 – 14 anos de idade (oitavo e nono ano). Primeiramente as escolas serão recrutadas via uma lista que será providenciada pelo Núcleo Regional de Educação de Jacarezinho, Pr, referente ao ano acadêmico de 2022. Posteriormente a aquisição da lista, o pesquisador responsável entrará em contato via e-mail com a equipe pedagógica (diretor e coordenador(s)) das escolas elegíveis convidando-as a participar do programa. Após o convite, será agendada uma reunião entre os representantes das escolas que demonstraram interesse e o coordenador da pesquisa, o qual terá como objetivo explicar e esclarecer alguns pontos e exigências do projeto.

Posteriormente a reunião, cada escola deverá providenciar uma lista com o número de alunos do oitavo e nono ano, onde uma classe de cada ano será selecionada por meio de uma randomização para participar do programa de atividade física. Em relação as disciplinas lecionadas (exemplo: educação física, matemática, português entre outras) não haverá nenhuma restrição.

Professores de educação física (ED.F) que concordar em inserir o programa de atividade física na aula de ED.F durante 4 meses e os estudantes que concordarem em participar do oitavo e nono ano. É importante enfatizar que os adolescentes que concordarem em participar do programa, também estarão concordando e consentindo a participar da avaliação dos componentes nos diferentes momentos. Para a avaliação, será indispensável a apresentação do termo de consentimento livre e esclarecido assinado tanto pelos pais ou responsáveis legais dos adolescentes como pelos próprios participantes autorizando o uso dos seus dados (Anexo I e II). Os adolescentes com diagnostico de doenças cardiometabólica (diabetes tipo 2; hipertensão) e com condição física e mental que venha a interferir em sua participação no programa será excluído do estudo (análise), porém ainda sim participara das aulas e da intervenção normalmente.

**Cálculo Amostral**

O tamanho amostral foi conduzido usando G*Power (versão 3.1) e com base nas mudanças do desfecho primário (atividade física). Com base em uma pesquisa prévia, um tamanho de efeito para AF de 0,25 e uma diferença ajustada de 5 minutos de AFMV por dia entre diferentes momentos (assumindo um desvio padrão de 17,8 minutos com correlação de 0,59 entre o baseline e pós-intervenção) foi considerada como acessível e clinicamente significante na população do estudo([36](#_ENREF_36)). Também foi ajustado por agrupamento no nível da classe usando o fator de correção [1 + (m - 1) x ICC], onde m representa o número de participantes por turma e ICC referente a coeficiente de correlação intra-classe para atividade física. Assumindo uma média de 29 adolescentes participantes, três classes por escolas e um ICC para atividade física de 0.034([37](#_ENREF_37)). Considerando uma perda de 20% da amostra no fim do estudo, 85% power com nível de alfa menor que 0.05, o tamanho amostral requerido é de 87 estudantes por escola.

A amostra estimada foi de 174 adolescentes de ambos os sexos (87 no grupo intervenção (GI) e 87 no grupo controle (GC)) que atendessem os seguintes critérios de inclusão: (I) entregar do TCLE e TALE devidamente assinado; (II) estar regularmente matriculado no 8º ou 9º ano do ensino fundamental II; (III) idade entre 13 e 14 anos. Os critérios de exclusão do estudo foram: (I) apresentar doença cardiometabólica reconhecida; (II) possuir algum tipo de doença genética, neoplásica ou mental que possa interferir na compreensão da atividade ou no desempenho durante a pesquisa; (III) não ter participado de todas as etapas do estudo. É importante ressaltar que os adolescentes com diagnostico de doenças cardiometabólica (diabetes tipo 2; hipertensão) e com condição física e mental que venha a interferir em sua participação no programa foram excluídos das análises do estudo, porém ainda sim participaram das aulas e da intervenção normalmente.

**Blindagem e Randomização**

A randomização irá ocorrer entre as escolas que forem recrutadas e que tenham completado as avaliações no baseline. As escolas serão randomizadas em intervenção ou controle por um pesquisador independente usando um software gerador de número aleatório por meio de um computador, e à randomização será realizada por bloco com base no nível socioeconômico da escola, por meio da classificação socioeconômica da Associação Brasileira de Empresa e Pesquisa ([38](#_ENREF_38)). As escolas randomizadas na condição intervenção terão o programa de atividade física durante o período do estudo, enquanto que as escolas do grupo controle continuará com a prática usual (aulas curriculares normais) durante o período do estudo (4 meses), e posteriormente a avaliação final, as escolas do controle receberão a intervenção.

**INTERVENTION**

O programa é uma intervenção de atividade física de base-escolar, o qual inclui o treinamento de resistência. Esta intervenção é uma adaptação do programa australiano Treinamento de Resistência para Jovens ([29](#_ENREF_29)), o qual é originário das intervenções NEAT e ATLAS ([39](#_ENREF_39)), e será designado para melhorar a aptidão física, saúde mental e cardiometabólica, e aumentar o nível de atividade física de adolescentes. Este programa multicomponente será desenvolvido durante o período escolar (16 semanas) nas aulas de educação física por aproximadamente 90 minutos semanais, ou seja, terá duração de 45 minutos por aula, e terá como alvo professores, escolas, estudantes e pais/responsáveis. A implementação das estratégias usadas para auxiliar a adoção incluirá: (i) workshop para os professores da rede básica pelos profissionais de pesquisa; (ii) entrega de um livro para os professores com informações sobre os recursos das sessões e o material ou equipamento necessário; e (iii) observação e feedback da sessão da atividade física. A intervenção consistirá nos seguintes componentes: (i) seminário introdutório para adolescentes realizados pelos professores; (ii) programa de atividade física estruturado, com base em treinamento de resistência por meio de cartão (educação física); (iii) pedômetro para o auto monitoramento e estabelecimento de metas (fora do ambiente escolar); (iv) envio de mensagens para estudantes e seus pais. Um resumo dos componentes pode ser verificado na tabela 1.

A intervenção será guiada pela teoria sócio-cognitiva (TSC)([40](#_ENREF_40)) e teoria da auto- determinação ([41](#_ENREF_41)), as sessões serão desenvolvidas para satisfazer as necessidades psicológicas básicas para a autonomia, competência, e relacionamento; motivação autônoma e auto eficácia para atividade física. Várias estratégias sociocultural ([42](#_ENREF_42)) serão aplicadas na intervenção para aumentar sua relevância e recurso para os adolescentes. Por exemplo, o cartão circuito e o seminário interativo incluirá imagens de modelos do sexo feminino e masculino. Em adição, o conteúdo do seminário interativo será elaborado levando em consideração tanto o gênero feminino como o masculino, para assim enfatizar a necessidade da mudança de comportamento da saúde. As sessões durante as aulas de educação física serão conduzidas com grupos misto (ambos os sexos).

A atividade física estruturada de treinamento de resistência (TR) ([29](#_ENREF_29), [39](#_ENREF_39)) consistirá de uma sessão específica incluindo: jogos com base em movimento e aquecimento muscular dinâmico; desenvolvimento das habilidades de TR; exercício com TR de alta intensidade (HIRT); jogos adaptados incluindo a aptidão, movimento de box, ou exercício de fortalecimento do core (abdominal); e alongamento estático. Participantes terão autonomia para selecionar seu próprio circuito, dentro de uma variedade de cartões de TR pré-designado, o qual será inserido de forma gradativa no decorrer do programa com o objetivo de promover uma variedade e manter o interesse/motivação do participante. A intensidade de cada sessão será verificada através da escala de percepção de BORG.

Para promover a aderência ao exercício, as sessões serão desenvolvidas com o objetivo de aprimorar a motivação autônoma do adolescente para TR ([29](#_ENREF_29), [39](#_ENREF_39)), tanto dentro como fora do contexto escolar, através de atividades que venham a estimular o sentimento de controle, capacidade e relacionamento social, os quais são princípios da teoria da auto-determinação ([41](#_ENREF_41)). Os professores aprenderão a facilitar as sessões de TR usando o princípio ensino SAAFE (suporte, ativo, autônomo, justo e prazeroso) ([43](#_ENREF_43)), que servirá como uma estrutura para o design e entrega das sessões de atividade física, bem como para as sessões de observação. Eles participarão de um workshop promovido pelos pesquisadores responsáveis, que terá como meta enfatizar a importância da aptidão muscular na saúde; providenciar estratégias de como inserir a aptidão muscular e o exercício de resistência nas aulas, e integrar o princípio SAAFE em suas aulas.

As necessidades dos participantes para autonomia serão promovidas pela oportunidade de escolha nas sessões (exemplo: tipo de atividade e preferência musical durante a prática) e pelo seminário introdutório informativo, o qual incluirá uma explicação e justificativa da importância do programa para a saúde física, perfil metabólico, saúde mental e mudança comportamental. A competência dos participantes será satisfeita por intermédio de feedback específico e positivo dos professores para enfatizar a auto-eficácia (providenciando encorajamento, dando feedback específico sobre a técnica e o desempenho correto do movimento) ([39](#_ENREF_39)). Professores serão encorajados adotar práticas que oportunize o relacionamento entre o grupo de alunos durante as sessões de TR([43](#_ENREF_43)). Em relação a pratica dos exercícios, algumas precauções serão consideradas para garantir a segurança do participante: 1) explicação e demonstração da técnica correta de todos os exercícios no seminário introdutório; 2) inclusão do aquecimento e da “volta a calma” em cada sessão; e 3) lembrete para professores e membros da pesquisa para monitorar e corrigir a técnica do exercício.

Estratégias adicionais usadas serão: pedômetro juntamente com estabelecimento de metas, onde cada adolescente do grupo intervenção (GI+P) receberá sua própria meta (baseado sobre o número de passos mensurado no baseline) que deve ser atingida semanalmente. Essas metas serão pré-determinadas por um pesquisador de maneira progressiva ([44](#_ENREF_44)), e também será enviado mensagens via WhatsApp® para estimular uma alimentação saudável e prática de atividade física regular tanto para os adolescentes da intervenção como para o grupo controle, e para o grupo dos pais/responsáveis ([45](#_ENREF_45)). Os adolescentes do grupo controle continuarão com a prática usual nas aulas de educação física e em práticas esportivas extracurriculares, sendo que receberão a intervenção após a avaliação final.

**Mensuração e coleta de dados**

Todas as avaliações serão conduzidas no ambiente escolar por pesquisadores assistente que serão blindados em relação a alocação do grupo na avaliação pós intervenção. Informações socioeconômicas e mensurações auto-reportada será avaliada por questionário, já a aptidão física será por testes. A mensuração antropométrica será conduzida por dois pesquisadores de ambos os sexos. O pesquisador assistente providenciara uma descrição verbal e demonstração do teste de aptidão antes do início da avaliação.

**Desfecho primário**

Atividade física (AF)

Os adolescentes serão instruídos a usar o acelerômetro GT3X no quadril na altura da espinha ilíaca ântero superior durante o dia todo (exceto no banho, quando for dormir ou em alguma atividade aquática) por um período de 7 dias consecutivos. Os sujeitos que usarem o tempo mínimo de > 8 horas por dia e mínimo de 3 dias (incluindo 1 dia de final de semana) será incluído na análise dos dados válidos. Será estimado o tempo (minutos/dia) gasto em atividade física nas diferentes intensidades (leve, moderado e vigoroso), por meio de um ponto de corte ([46](#_ENREF_46)). O tempo de não uso, definido como ≥30 minutos de “0” counts contínuos, será removido da análise ([47](#_ENREF_47)). Os dados referentes aos dias de semana e final de semana serão calculados separadamente.

**Desfechos secundários**

Aptidão muscular

A resistência muscular de membros superiores e interiores será mensurada através do teste de flexão de 90º graus([48](#_ENREF_48)) e sentar- levantar([49](#_ENREF_49)), respectivamente. No teste de flexão de 90 graus, o participante deverá realizar uma flexão até formar um ângulo de 90 graus de cotovelo e posteriormente realiza a extensão de cotovelo, dentro de uma cadência de 40 *“beep*” por minutos. O teste será encerrado quando o participante falhar em realizar uma flexão dentro do ângulo exigido em duas repetições não consecutivas (o avaliador informará sobre o erro e as repetições são contadas em voz alta), falhar em manter as repetições dentro do metrônomo, falhar em manter a técnica apropriada (coluna ereta) ou na falha volitiva do teste. Para o membro inferior, sentará em uma cadeira (ajustável na altura do participante) com suas costas apoiada na cadeira. Os voluntários serão orientados a ir da posição sentada para a em pé e da posição em pé para sentada por maior número de vezes possível de maneira rítmica, dentro de um período de 30 segundos. Este teste será desenvolvido duas vezes, com um intervalo de 3 minutos entre eles, e o maior número de repetição será utilizado como repetição final.

Saúde cardiometabólica

As variáveis cardiometabólicas que serão analisadas são glucose, insulina, triglicerídeo (TG), colesterol total (CT), hemoglobina glicada (HbA1c). Amostras de sangue serão coletados da veia cubital em tubos a vácuo após 12 horas em jejum, em dois momentos diferentes (baseline e pós-intervenção), seguindo a recomendação da Sociedade Brasileira de Patologia Clínica/Medicina Laboratorial. A glicose em jejum será mensurada usando a Hexocinase Enzimática. Para determinar a insulina em jejum será usado o método Quimioluminescencia. O colesterol total e os triglicerídeos serão analisados pelo método Colorimetrico Enzimático, e hemoglobina glicada será determinada usando Cromatrografia Líquida de Alta Eficiência. A avaliação do modelo de homeostase para resistência à insulina (HOMA-IR) será usado para determinar a sensibilidade da insulina, e será calculado usando a seguinte fórmula: [(glicose*0,0556)*insulin]/22,5 ([50](#_ENREF_50)).

Aptidão cardiorrespiratória (APR)

A aptidão cardiorrespiratória será avaliada pelo teste PACER Fitnessgram e será administrado seguindo os procedimentos padronizados([51](#_ENREF_51)), o qual tem uma excelente validade e confiabilidade nesta população ([52](#_ENREF_52)). Os avaliadores providenciarão encorajamento verbal durante o teste para maximizar a motivação do participante. Uma distância de 20 metros será demarcada com cones em uma superfície plana, os participantes serão instruídos a ir de um ponto demarcado ao outro ponto mantendo um ritmo e uma cadência pré-determinada (indicada por um sinal sonoro conhecido como “*beep*”). O teste inicia a 8.5km/h e aumenta 0.5 km/h a cada minuto, e o teste é encerrado quando o participante falha em completar duas voltas consecutivas no tempo pré-determinado ou quando tem a desistência voluntária devido a fadiga. O último estágio válido anotado e convertido no número de voltas completas de 20m, e o número total de voltas será usado para estimar a capacidade aeróbico máxima (VO_2_max) através da equação: 45.619+(0.353*PACER laps)–(1.121*age) ([53](#_ENREF_53)).

Saúde Mental

Dificuldades psicológicas

O “questionário de pontos fortes e dificuldades” será usado para avaliar problemas psicológicos dos adolescentes ([54](#_ENREF_54), [55](#_ENREF_55)). Este instrumento consiste em 25 itens, subdividido sub-itens:1) pontos fortes, que é composto de um domínio (comportamento pró-social) e 2) dificuldades, composto de 4 domínios (sintomas emocionais, conduta problemática, hiperatividade e problemas com o colega). Para cada domínio, o adolescente deverá responder cada item utilizando uma escala de 0 (não é verdadeiro) à 2 (certamente verdadeiro), ou seja, a pontuação pode variar de 0 a 10 em cada domínio e escore baixos indicam menor problema/dificuldade psicológica.

Bem-estar

A mensuração da variável psicológica “Bem-Estar” será por e dois domínios do questionário KIDSCREEN-27, é um instrumento auto relatado que consiste de 5 item relacionado com bem-estar físico e 7 itens relacionado ao bem –estar psicológico. Cada ítem é composta de uma escala Likert de 5 pontos([56](#_ENREF_56)).

Sono

A qualidade e a quantidade do sono serão avaliados pelo Índice de Qualidade do Sono de Pittsburgh (PSQI)([57](#_ENREF_57)), validado para adolescentes brasileiros([58](#_ENREF_58)). O PSQI é um questionário autoaplicável que pede aos respondentes que relatem a qualidade do sono e os sinais de distúrbios do sono no período de 1 mês anterior ao preenchimento do questionário. O PSQI inclui 19 questões, categorizadas em sete grupos (qualidade do sono, latência do sono, duração do sono, eficiência habitual do sono, distúrbios do sono, uso de medicamentos para dormir e disfunção diurna). Cada questão constituinte produz uma pontuação em uma escala do tipo Likert de 4 pontos (de 0 a 3) e a pontuação total é composta de pontuações de cada um dos sete subgrupos de perguntas, dando uma pontuação acumulada entre 0 e 21.

Composição corporal

Serão realizadas medidas de massa corporal, estatura e circunferência de cintura em uma sala reservada com dois avaliadores de ambos os sexos para evitar qualquer tipo de constrangimento do voluntário que estará sendo avaliado. A estatura será medida através de um estadiômetro vertical portátil (Welmy®, Santa Bárbara do Oeste, São Paulo, Brasil), escalonado em 0,1 cm massa e a massa corporal será mensurada utilizando uma balança digital portátil (Welmy®, Santa Bárbara do Oeste, São Paulo, Brasil), com resolução de 0,1kg. Ambas mensurações serão realizadas por duas vezes com o objetivo de reduzir o erro técnico de mensuração. Uma terceira avaliação será realizada caso a diferença entre a primeira e a segunda medida forem maiores que 0.1 kg para massa e 0.3 cm para altura. Diante destas informações, o índice de massa corporal (IMC) será calculado usando a equação padrão (peso[kg]/estatura^2^[cm]) e o IMC z-score será determinado usando o método ‘LMS’ de acordo com os dados da Organização Mundial da Saúde([59](#_ENREF_59)).

A circunferência de cintura será aferida no ponto médio entre o último arco costal e a crista ilíaca, utilizando uma fita antropométrica inextensível (Sanny®, São Bernardo do Campo, São Paulo, Brasil).

**Mediadores Hipotetizados**

Auto-eficácia para treinamento de resistência

A auto-eficácia será avaliada por meio de um questionário desenvolvido especificamente para adolescentes([60](#_ENREF_60)). Este questionário é composto por 4 itens e avalia o nível de confiança dos adolescentes em se engajar no treinamento de resistência e as respostas das questões variam em uma escala tipo Likert de 5 pontos (1= discordo totalmente para 5= concordo totalmente).

Satisfação psicológica básica necessária

O “questionário suporte psicológico necessário para exercício em adolescentes’ será usado para avaliar o suporte dos professores e dos amigos para o exercício ([61](#_ENREF_61)). A avaliação da satisfação durante o exercício inclui 3-itens: suporte autônomo, suporte do relacionamento e a competência deste suporte. O adolescente deverá responder a sua satisfação em cada item usando uma escala Likert que varia entre 1 (discordo totalmente) e 7 (concordo totalmente).

Motivação autônoma

A motivação autônoma para atividade física será avaliada por meio do ‘questionário de regulação comportamental no exercício’([62](#_ENREF_62)), o qual é composto por duas sub-escalas: regulação intrínseca e identificada, sendo que cada escala varia entre 0 ( não é verdadeira para mim) e 4 (muito verdadeiro para mim).

**Variáveis de controle**

Maturação somática

A maturação somática de cada adolescente será estimada por meio da equação do pico de velocidade em crescimento([63](#_ENREF_63)), específica por sexo, que inclui a estatura, altura sentado, comprimento da perna, massa corporal, idade cronológica e suas interações. O comprimento da perna será calculado subtraindo a estatura sentada da estatura em pé.

**Avaliação do processo**

A viabilidade do programa será avaliada baseadas nos seguintes critérios: 1) taxa de consentimento (qual o número de adolescentes que concordaram em participar do programa, após o mesmo ter sido ofertado); 2) taxa de retenção (referente aos 4 meses de seguimento); 3) frequência (participação dos estudantes na atividade física estruturada); 4) satisfação dos estudantes com o programa ( “ eu gostei de participar das sessões de treinamento de resistência” e a resposta varia entre 5 (concordo totalmente) e 1 (descordo totalmente) e 5) engajamento ( o uso do pedômetro e aderência as metas estabelecidas).

**Análise estatística**

Os dados obtidos serão tabulados no programa Excel (Microsoft Windows, USA, 2013) e analisados utilizando-se o pacote estatístico Statistical Package for the Social Sciences versão 20.0 (SPSS, USA, 2012), sendo adotado um nível de significância de p < 0,05. O efeito da intervenção nas variáveis desfecho primário e secundário serão analisados através da do modelo linear misto, considerando-se os fatores (controle, intervenção e intervenção com pedômetro) e tempo (linha de base e após o protocolo). Para complementar as análises o tamanho do efeito (η²) será apresentado. Potenciais moderadores serão explorados usando os modelos lineares mistos com interação para sexo (feminino ou masculino), nível socioeconômico (baixo, médio e alto), estado do peso (saudável vs. sobrepeso/obeso). Análises dos subgrupos serão conduzidas somente se o efeito da interação for P≤0.10.

Para analisar os mediadores hipotetizados, será utilizado a regressão linear usando a extensão PROCESS macro para Windows (HAYES, 2013).

RESULTADOS ESPERADOS

Considerando os diversos benefícios promovidos pela prática regular de atividade física relatados na literatura, espera-se que o programa de promoção de AF aumente os níveis de atividade física dos adolescentes e, consequentemente, promova melhoras nos indicadores de aptidão física, saúde cardiometabólica e mental.

Referência:

1. Hallal PC, Victora CG, Azevedo MR, Wells JC. Adolescent physical activity and health: a systematic review. Sports medicine (Auckland, NZ). 2006;36(12):1019-30.

2. WHO. WHO Guidelines Approved by the Guidelines Review Committee. Global Recommendations on Physical Activity for Health. Geneva: World Health Organization

Copyright (c) World Health Organization 2010.; 2010.

3. Rhodes RE, Janssen I, Bredin SSD, Warburton DER, Bauman A. Physical activity: Health impact, prevalence, correlates and interventions. Psychology & Health. 2017;32(8):942-75.

4. Werneck AO, Oyeyemi AL, Fernandes RA, Romanzini M, Ronque ERV, Cyrino ES, et al. Regional Socioeconomic Inequalities in Physical Activity and Sedentary Behavior Among Brazilian Adolescents. Journal of physical activity & health. 2018;15(5):338-44.

5. Silva PRd, Santos GCd, Faria WFd, Corrêa RC, Elias RGM, Stabelini Neto A. Tracking of physical activity in adolescents between 2010 and 2014. Revista Brasileira de Cineantropometria & Desempenho Humano. 2018;20(1):64-70.

6. Dumith SC, Gigante DP, Domingues MR, Kohl HW, III. Physical activity change during adolescence: a systematic review and a pooled analysis. International Journal of Epidemiology. 2011;40(3):685-98.

7. Boddy LM, Thomas NE, Fairclough SJ, Tolfrey K, Brophy S, Rees A, et al. ROC Generated Thresholds for Field-Assessed Aerobic Fitness Related to Body Size and Cardiometabolic Risk in Schoolchildren. PLOS ONE. 2012;7(9):e45755.

8. Ross R, Blair SN, Arena R, Church TS, Despres JP, Franklin BA, et al. Importance of Assessing Cardiorespiratory Fitness in Clinical Practice: A Case for Fitness as a Clinical Vital Sign: A Scientific Statement From the American Heart Association. Circulation. 2016;134(24):e653-e99.

9. Smith JJ, Morgan PJ, Plotnikoff RC, Dally KA, Salmon J, Okely AD, et al. Smart-phone obesity prevention trial for adolescent boys in low-income communities: the ATLAS RCT. Pediatrics. 2014;134(3):e723-31.

10. Ortega FB, Artero EG, Ruiz JR, Vicente-Rodriguez G, Bergman P, Hagstromer M, et al. Reliability of health-related physical fitness tests in European adolescents. The HELENA Study. International journal of obesity (2005). 2008;32 Suppl 5:S49-57.

11. Collings PJ, Westgate K, Vaisto J, Wijndaele K, Atkin AJ, Haapala EA, et al. Cross-Sectional Associations of Objectively-Measured Physical Activity and Sedentary Time with Body Composition and Cardiorespiratory Fitness in Mid-Childhood: The PANIC Study. Sports medicine (Auckland, NZ). 2017;47(4):769-80.

12. Marques A, Santos R, Ekelund U, Sardinha LB. Association between physical activity, sedentary time, and healthy fitness in youth. Medicine and science in sports and exercise. 2015;47(3):575-80.

13. Prevention. CfDCa. Comprehensive school physical activity programs: a guide for schools. In: Services UDoHaH, editor. Atlanta, GA2013.

14. Tremblay MS, Barnes JD, Gonzalez SA, Katzmarzyk PT, Onywera VO, Reilly JJ, et al. Global Matrix 2.0: Report Card Grades on the Physical Activity of Children and Youth Comparing 38 Countries. Journal of physical activity & health. 2016;13(11 Suppl 2):S343-s66.

15. Silva DAS, Chaput JP, Tremblay MS. Participation frequency in physical education classes and physical activity and sitting time in Brazilian adolescents. PLoS One. 2019;14(3):e0213785.

16. Telford RD, Cunningham RB, Fitzgerald R, Olive LS, Prosser L, Jiang X, et al. Physical education, obesity, and academic achievement: a 2-year longitudinal investigation of Australian elementary school children. American journal of public health. 2012;102(2):368-74.

17. Standal ØF, Aggerholm K. Habits, skills and embodied experiences: a contribution to philosophy of physical education. Sport, Ethics and Philosophy. 2016;10(3):269-82.

18. Hollis JL, Williams AJ, Sutherland R, Campbell E, Nathan N, Wolfenden L, et al. A systematic review and meta-analysis of moderate-to-vigorous physical activity levels in elementary school physical education lessons. Preventive Medicine. 2016;86:34-54.

19. da Costa BGG, da Silva KS, da Silva JA, Minatto G, de Lima LRA, Petroski EL. Sociodemographic, biological, and psychosocial correlates of light- and moderate-to-vigorous-intensity physical activity during school time, recesses, and physical education classes. Journal of sport and health science. 2019;8(2):177-82.

20. Buchan DS, Ollis S, Thomas NE, Buchanan N, Cooper S-M, Malina RM, et al. Physical activity interventions: effects of duration and intensity. Scandinavian journal of medicine & science in sports. 2011;21(6):e341-e50.

21. Love R, Adams J, van Sluijs EMF. Are school-based physical activity interventions effective and equitable? A meta-analysis of cluster randomized controlled trials with accelerometer-assessed activity. Obesity Reviews. 2019;20(6):859-70.

22. Dobbins M, Husson H, DeCorby K, LaRocca RL. School-based physical activity programs for promoting physical activity and fitness in children and adolescents aged 6 to 18. The Cochrane database of systematic reviews. 2013(2):Cd007651.

23. Bauman AE, Reis RS, Sallis JF, Wells JC, Loos RJF, Martin BW, et al. Correlates of physical activity: why are some people physically active and others not? Lancet. 2012;380(9838):258-71.

24. Martins J, Marques A, Sarmento H, Carreiro da Costa F. Adolescents' perspectives on the barriers and facilitators of physical activity: a systematic review of qualitative studies. Health Educ Res. 2015;30(5):742-55.

25. Corder K, Atkin AJ, Ekelund U, van Sluijs EM. What do adolescents want in order to become more active? BMC public health. 2013;13:718.

26. Hulteen RM, Smith JJ, Morgan PJ, Barnett LM, Hallal PC, Colyvas K, et al. Global participation in sport and leisure-time physical activities: A systematic review and meta-analysis. Prev Med. 2017;95:14-25.

27. Lubans DR, Morgan PJ, Okely AD, Dewar D, Collins CE, Batterham M, et al. Preventing Obesity Among Adolescent Girls: One-Year Outcomes of the Nutrition and Enjoyable Activity for Teen Girls (NEAT Girls) Cluster Randomized Controlled Trial. Archives of pediatrics & adolescent medicine. 2012;166(9):821-7.

28. Dewar DL, Morgan PJ, Plotnikoff RC, Okely AD, Collins CE, Batterham M, et al. The nutrition and enjoyable activity for teen girls study: a cluster randomized controlled trial. American journal of preventive medicine. 2013;45(3):313-7.

29. Kennedy SG, Smith JJ, Morgan PJ, Peralta LR, Hilland TA, Eather N, et al. Implementing Resistance Training in Secondary Schools: A Cluster Randomized Controlled Trial. Medicine and science in sports and exercise. 2018;50(1):62-72.

30. Smith JJ, Morgan PJ, Plotnikoff RC, Dally KA, Salmon J, Okely AD, et al. Smart-phone obesity prevention trial for adolescent boys in low-income communities: the ATLAS RCT. Pediatrics. 2014;134(3):e723-e31.

31. Lubans DR, Smith JJ, Morgan PJ, Beauchamp MR, Miller A, Lonsdale C, et al. Mediators of Psychological Well-being in Adolescent Boys. The Journal of adolescent health : official publication of the Society for Adolescent Medicine. 2016;58(2):230-6.

32. Filho VCB, Silva KSd, Mota J, Beck C, Lopes AdS. A Physical Activity Intervention for Brazilian Students From Low Human Development Index Areas: A Cluster-Randomized Controlled Trial. 2016;13(11):1174.

33. Costa B, Silva KSD, Silveira PMD, Berria J, Machado AR, Petroski EL. The effect of an intervention on physical activity of moderate-and-vigorous intensity, and sedentary behavior during adolescents' time at school. Brazilian journal of epidemiology

2019;22:e190065.

34. Leme AC, Lubans DR, Guerra PH, Dewar D, Toassa EC, Philippi ST. Preventing obesity among Brazilian adolescent girls: Six-month outcomes of the Healthy Habits, Healthy Girls-Brazil school-based randomized controlled trial. Prev Med. 2016;86:77-83.

35. Moher D, Hopewell S, Schulz KF, Montori V, Gotzsche PC, Devereaux PJ, et al. CONSORT 2010 explanation and elaboration: updated guidelines for reporting parallel group randomised trials. BMJ (Clinical research ed). 2010;340:c869.

36. Corder K, Brown HE, Schiff A, van Sluijs EMF. Feasibility study and pilot cluster-randomised controlled trial of the GoActive intervention aiming to promote physical activity among adolescents: outcomes and lessons learnt. BMJ open. 2016;6(11):e012335.

37. Corder K, Sharp SJ, Atkin AJ, Griffin SJ, Jones AP, Ekelund U, et al. Change in objectively measured physical activity during the transition to adolescence. Br J Sports Med. 2015;49(11):730-6.

38. ABEP. Associação Brasileira de Empresas de Pesquisa - Critério Brasil: Estrato Sócio Econômico

2019.

39. Lubans DR, Smith JJ, Peralta LR, Plotnikoff RC, Okely AD, Salmon J, et al. A school-based intervention incorporating smartphone technology to improve health-related fitness among adolescents: rationale and study protocol for the NEAT and ATLAS 2.0 cluster randomised controlled trial and dissemination study. BMJ open. 2016;6(6):e010448.

40. Bandura A. Health promotion by social cognitive means. Health education & behavior : the official publication of the Society for Public Health Education. 2004;31(2):143-64.

41. Deci EL, Ryan RM. The "What" and "Why" of Goal Pursuits: Human Needs and the Self-Determination of Behavior. Psychological Inquiry. 2000;11(4):227-68.

42. Morgan PJ, Young MD, Smith JJ, Lubans DR. Targeted Health Behavior Interventions Promoting Physical Activity: A Conceptual Model. Exercise and sport sciences reviews. 2016;44(2):71-80.

43. Lubans DR, Lonsdale C, Cohen K, Eather N, Beauchamp MR, Morgan PJ, et al. Framework for the design and delivery of organized physical activity sessions for children and adolescents: rationale and description of the 'SAAFE' teaching principles. The international journal of behavioral nutrition and physical activity. 2017;14(1):24.

44. Kantanista A, Bronikowski M, Laudanska-Krzeminska I, Krol-Zielinska M, Osinski W. Positive effect of pedometer-based walking intervention on body image and physical activity enjoyment in adolescent girls. Biomedical Human Kinetics. 2017;9(1):34-42.

45. Service HP. Parent Guide: how you can support physical activity and weelbeing for your tennegers. In: Health Promotion Service- Directorate of Planning PHaE, editor. South Eastern Sydney Local Health District, Australia2016.

46. Evenson KR, Catellier DJ, Gill K, Ondrak KS, McMurray RG. Calibration of two objective measures of physical activity for children. Journal of sports sciences. 2008;26(14):1557-65.

47. Masse LC, Fuemmeler BF, Anderson CB, Matthews CE, Trost SG, Catellier DJ, et al. Accelerometer data reduction: a comparison of four reduction algorithms on select outcome variables. Medicine and science in sports and exercise. 2005;37(11 Suppl):S544-54.

48. Cooper. Fitnessgram: Test administration manual. Champaign, IL: Human Kinetics1999.

49. Bohannon RW. Sit-to-stand test for measuring performance of lower extremity muscles. Perceptual and motor skills. 1995;80(1):163-6.

50. Oliveira EPd, Souza MLAd, Lima MdDAd. Índice HOMA (homeostasis model assessment) na prática clínica: uma revisão. Jornal Brasileiro de Patologia e Medicina Laboratorial. 2005;41:237-43.

51. Meredith MD, GJ W. FitnessGram & ActivityGram test administration manual. Champaign: IL: Human Kinetics. 2010;Update 4 th ed.

52. Lang JJ, Tomkinson GR, Janssen I, Ruiz JR, Ortega FB, Leger L, et al. Making a Case for Cardiorespiratory Fitness Surveillance Among Children and Youth. Exercise and sport sciences reviews. 2018;46(2):66-75.

53. Mahar MT, Welk GJ, Rowe DA. Estimation of aerobic fitness from PACER performance with and without body mass index. Measurement in Physical Education and Exercise Science. 2018;22(3):239-49.

54. Goodman R. Psychometric Properties of the Strengths and Difficulties Questionnaire. Journal of the American Academy of Child & Adolescent Psychiatry. 2001;40(11):1337-45.

55. Fleitlich-Bilyk B, Goodman R. Prevalence of Child and Adolescent Psychiatric Disorders in Southeast Brazil. Journal of the American Academy of Child & Adolescent Psychiatry. 2004;43(6):727-34.

56. Farias Júnior JCd, Loch MR, Lima Neto AJd, Sales JM, Ferreira FELdL. Reprodutibilidade, consistência interna e validade de construto do KIDSCREEN-27 em adolescentes brasileiros. Cadernos de Saúde Pública. 2017;33:e00131116.

57. Buysse DJ, Reynolds CF, 3rd, Monk TH, Berman SR, Kupfer DJ. The Pittsburgh Sleep Quality Index: a new instrument for psychiatric practice and research. Psychiatry research. 1989;28(2):193-213.

58. Passos MH, Silva HA, Pitangui AC, Oliveira VM, Lima AS, Araújo RC. Reliability and validity of the Brazilian version of the Pittsburgh Sleep Quality Index in adolescents. J Pediatr (Rio J). 2017;93(2):200-6.

59. WHO. WHO Multicentre Growth Reference Study Group. WHO Growth reference data 5-19 years: BMI-for-age (5-19 years). Geneva: World Health Organization. 2007.

60. Lubans DR, Morgan P, Callister R, Plotnikoff RC, Eather N, Riley N, et al. Test-retest reliability of a battery of field-based health-related fitness measures for adolescents. Journal of sports sciences. 2011;29(7):685-93.

61. Emm-Collison LG, Standage M, Gillison FB. Development and Validation of the Adolescent Psychological Need Support in Exercise Questionnaire. J Sport Exerc Psychol. 2016;38(5):505-20.

62. Markland D, Tobin V. A modification to the Behavioural Regulation in Exercise Questionnaire to include an assessment of amotivation. Journal of sport & exercise psychology. 2004;26(2):191-6.

63. Mirwald RL, Baxter-Jones ADG, Bailey DA, Beunen GP. An assessment of maturity from anthropometric measurements. Med Sci Sports Exerc. 2002;34(4):689-94.

Anexo I - TERMO DE CONSENTIMENTO LIVRE E ESCLARECIDO – TCLE

TERMO DE CONSENTIMENTO LIVRE ESCLARECIDO

Prezado(a) Senhor(a):

a) Seu filho (a) está sendo convidado a participar de um estudo intitulado “Efeitos de um programa de promoção da atividade física no nível de atividade física, aptidão física, saúde cardiometabólica e mental em adolescentes”.

b) O objetivo deste estudo é avaliar os efeitos de um programa de promoção da atividade física sobre o nível de atividade física, os músculos, condicionamento físico, níveis de glicose (açúcar no sangue) e lipídios (gordura no sangue), e mental (sinais e sintomas de ansiedade, estresse, depressão, relacionamento com os colegas) em adolescentes.

c) A participação do seu filho (a) é muito importante, pois ele atende os critérios de inclusão do estudo, os quais abrangem: estar regularmente matriculado no 8º ou 9º ano do ensino fundamental II; idade entre 13 e 14 anos; entregar o termo de consentimento livre e esclarecido assinado pelo responsável (TCLE) e o termo de assentimento livre e esclarecido assinado pelo participante (TALE). Assim que o senhor (a) autorizar a participação do seu filho (a), o mesmo realizará avaliações da seguinte forma: responderá a uma anamnese (nome; data de nascimento; nome dos responsáveis); questionário de atividade física. Posteriormente, será submetido a medidas do seu peso, altura, circunferências da cintura e quadril, testes motores e análise de sangue no início da pesquisa e após 4 meses e um ano após o fim da intervenção. Quanto a retirada do sangue, o mesmo será realizada por uma enfermeira registrada e posteriormente encaminhado ao laboratório contratado de análises clínicas em Jacarezinho-Pr, as amostras de sangue serão descartadas logo após a realização da análise sanguínea. Logo após a avaliação inicial, os participantes serão distribuídos de forma aleatória (sorteio) em grupo experimental ou controle. Os participantes do grupo experimental receberão um programa de intervenção imediatamente após a avaliação inicial, enquanto que os participantes do grupo controle receberão o programa de intervenção logo após a avaliação do seguimento.

d) O programa de intervenção que seu filho (a) participará terá duração de 4 meses composto por uma sessão de atividade física estruturada, auto-monitoramento e orientação sobre um estilo de vida saudável. A sessão de atividade física estruturada ocorrerá durante as aulas de educação física escolar sob a supervisão dos pesquisadores auxiliares, o qual incluirá exercícios para melhorar a força/resistência muscular e o condicionamento cardiorrespiratório. Em relação ao auto-monitoramento, o participante receberá um instrumento (aparelho) chamado pedômetro, que tem o objetivo de marcar o número de passos por dia realizado pelo indivíduo auxiliando no auto-monitoramento para adquirir um estilo de vida ativo. Este aparelho deverá ser colocado na altura do quadril (colocado no cós da calça/bermuda/short) e deverá ser utilizado diariamente ao longo do dia, exceto para dormir, nadar e tomar banho. Ao retirar o adolescente deverá anotar o número de passos acumulado durante o dia em um diário que também será entregue junto ao aparelho. Orientação sobre um estilo de vida saudável, serão enviadas mensagens pelo aplicativo WhatsApp® para incentivar um comportamento saudável. Após os 4 de intervenção e um ano pós intervenção, todos os participantes participarão das mesmas avaliações realizadas no início do projeto.

e) Estão garantidas todas as informações que você queira, antes, durante e após o estudo. Vale ressaltar que o participante terá acesso aos resultados dos exames laboratoriais e resultado final da pesquisa, se assim desejar.

f) Caso o seu filho não atinja o resultados esperado, encaminharemos para a Estratégia e Saúde da Família (ESF).

g) A participação de seu filho (a) é voluntária. Você tem a liberdade de recusar a participar do estudo, ou retirar seu consentimento a qualquer momento. Além disso, participante tem o direto de desistir da pesquisa a qualquer momento, sem que isso acarrete algum prejuízo.

h) Todas as despesas necessárias para a realização da pesquisa não são da responsabilidade do participante ou do seu responsável, e sim dos pesquisadores responsáveis. Caso o participante tiver alguma despesa referente à participação na pesquisa, o mesmo terá direito ao ressarcimento, custeado pela equipe de pesquisa.

i) Os adolescentes serão informados sobre os riscos do comportamento sedentários. Os benefícios esperados com a intervenção são a mudança de comportamento no estilo de vida, os quais incluem: Aumento do nível de atividade física dos adolescentes; redução dos níveis de açúcar e gordura no sangue; melhora da musculatura e do condicionamento físico (cardiorrespiratório), diminuição do comportamento sedentário; hábitos alimentares, controle psicológico (autocontrole; automotivação; relacionamento interpessoal).

j) Não são previstos riscos ou desconfortos inaceitáveis à participação no estudo. Os riscos são mínimos. Em relação a avaliação, todos os questionários serão aplicados de forma auto relatado, ou seja, o participante responderá individualmente e de acordo com a sua própria interpretação. Assim, o adolescente NÃO será exposto ou comparado com seus colegas evitando qualquer tipo de constrangimento e transtornos psicológicos durante a avaliação. Dores musculares devido aos testes físicos, todos os testes serão conduzidos e supervisionados por pesquisadores experientes que demonstrarão aos participantes como deverá ser executado os movimentos, minimizando assim qualquer tipo de lesão por execução de movimento errado. Quanto a coleta sanguínea, os riscos também são mínimos (dores leves na hora da pulsão sanguínea), pois a coleta será realizada em local apropriado, por pessoas especializadas (enfermeiros), porém caso ocorra algum incidente o adolescente juntamente com seu responsável (que deverá estar presente no dia da coleta) serão levados imediatamente a Santa Casa de Misericórdia de Jacarezinho. Em relação a intervenção, poderá ocorrer possíveis dores musculares devido aos exercícios inseridos durante a aula de educação física e ao aumento do número de passos (intervenção da atividade física). No entanto, para aliviar e ou minimizar esse desconforto serão realizados aquecimento muscular antes de iniciar o exercício e alongamento ao final de cada sessão; também será respeitado o tempo de descanso muscular (48 horas pós atividade). Além disso, os adolescentes serão orientados quando a adotar uma alimentação saudável e hidratação diária.

h) Quando os resultados forem publicados, não aparecerá o nome dos participantes, e sim um código.

i) O participante tem direito à indenização por danos decorrentes da pesquisa, nos termos da lei.

j) O termo de consentimento livre e esclarecido (TCLE) será redigido em duas vias, rubricado em todas as páginas e assinado pelo pesquisador, sendo uma via para o pesquisador e a outra do participante.

l) Caso tenha dúvidas ou necessite de maiores esclarecimentos sobre a pesquisa poderá entrar em contato com os pesquisadores (Antonio Stabelini Neto, endereço: Rua Alvaro Brochado - nº1108; Bairro: Nova Jacarezinho, Jacarezinho-Pr, CEP: 86400-000; Telefone: (43)3525-7357; Email: asneto@uenp.edu.br. Géssika Castilho dos Santos, endereço: Alameda Padre Magno, nº841; Bairro: Nova Jacarezinho, CEP: 86400-000, Jacarezinho-PR; Telefone: (43) 3525-0498 e (43) 9900-0265; email: gessika.castilho@gmail.com), ou procurar o Comitê de Ética em Pesquisa da Universidade Estadual do Norte do Paraná (CEP/UENP), telefone: (43) 3542-8056, situado no endereço: Rodovia BR 369 Km 54 - Universidade Estadual do Norte do Paraná - UENP. Bandeirantes - Pr. Este termo deverá ser preenchido em duas vias de igual teor, sendo uma delas, devidamente preenchida e assinada entregue a você.

Eu, ___________________________________________________________ li e concordo que meu meu filho (a)_______________________________________________________ participe da pesquisa.

____________________________________________

Assinatura do responsável ou impressão datiloscópica

Data: ___/___/____

Eu, Antonio Stabelini Neto, declaro que forneci todas as informações referentes ao projeto de pesquisa supra-nominado.

____________________________________________

Antonio Stabelini Neto

Data: ___/___/____

Eu, Géssika Castilho dos Santos, declaro que forneci todas as informações referentes ao projeto de pesquisa supra-nominado.

____________________________________________

Géssika Castilho dos Santos

Data: ___/___/____

Jacarezinho, ___ de ________de 202_.

Anexo II: TERMO DE ASSENTIMENTO LIVRE E ESCLARECIDO – TALE

TERMO DE ASSENTIMENTO LIVRE E ESCLARECIDO

O que significa assentimento?

O assentimento significa que você concorda em fazer parte de um grupo de adolescentes, da sua faixa de idade, para participar de uma pesquisa. Serão respeitados seus direitos e você receberá todas as informações por mais simples que possam parecer.

Pode ser que este documento denominado TERMO DE ASSENTIMENTO LIVRE E ESCLARECIDO contenha palavras que você não entenda. Por favor, peça ao responsável pela pesquisa ou à equipe do estudo para explicar qualquer palavra ou informação que você não entenda claramente.

Prezado(a) adolescente:

Você está sendo convidado para participar da pesquisa “Efeitos de um programa de promoção da atividade física no nível de atividade física, aptidão física, saúde cardiometabólica e mental em adolescentes”. Seus pais permitiram que você participe.

Queremos saber se o programa de atividade física promoverá uma melhora na força/resistência muscular e no condicionamento físico, melhora no nível de açúcar e gordura no sangue, melhora nos níveis de ansiedade, estresse, interação social e aumento na atividade física, e os adolescentes que irão participar dessa pesquisa têm de 13 a 14 anos de idade. Você não precisa participar da pesquisa se não quiser, é um direito seu, não terá nenhum problema se desistir. A pesquisa será realizada na escola durante as aulas de educação física, onde os participantes participarão da seguinte forma:

- Primeiramente o participante :

1) Responderá a um questionário sobre informações pessoais; nível socioeconômico e um questionário sobre os determinantes da atividade física. Serão submetido a avaliações de atividade física, testes físicos, responder questionários, medidas do seu peso, altura, circunferências da cintura, e análise de sangue. A retirada do sangue será realizada 3 vezes em momentos diferentes por uma enfermeira registrada e logo após encaminhado ao laboratório particular de análises clínicas em Jacarezinho-Pr. Assim que a análise sanguínea for realizada, sua amostra de sangue será descartada. As avaliações ocorrerão em 3 momentos diferentes : início e final da pesquisa, e após 1 ano .

- Após a avaliação inicial:

2) Os participantes serão distribuídos por meio de um sorteio em grupo experimental ou controle, ou seja, você poderá ser do grupo experimental ou controle. Todos receberão a intervenção. Os participantes do grupo experimental participarão primeiro do programa de intervenção de atividade física durante 4 meses, o qual é composto por uma sessão de atividade física estruturada, uso de um aparelho para monitorar o número de passos por dia, e orientação sobre um estilo de vida ativo. Enquanto que os participantes que estiverem no grupo controle irão participar da intervenção um ano após o fim da intervenção do grupo experimental.

3) Você participará de sessão estruturada nas aulas de educação físicas durante 4 meses, o qual cada sessão terá duração de 20 minutos. Nesta sessão de atividade física, você irá escolher qual os exercícios que deseja realizar, os quais incluem: fortalecimento muscular (por exemplo: agachamento, flexão de braço, abdominal) e de condicionamento cardiorrespiratório (por exemplo: polichinelo, pular corda). Durante toda a sessão você será supervisionado pelo seu professor de educação física e por um pesquisador auxiliar/ajudante. Vocês também receberão um aparelho chamado pedômetro para o auxiliar no monitoramento do número de passos realizado diariamente. Este aparelho deverá ser colocado na altura do quadril (colocado no cós da calça/bermuda/short) e utilizado ao longo do dia, exceto para dormir, nadar e tomar banho. Este aparelho deverá ser colocado de manhã e retirado na hora de dormir, e ao retirar o adolescente deverá anotar o número de passos acumulado durante o dia em um diário que também será entregue junto ao aparelho. Você também receberá mensagens semanais através do aplicativo WhatsApp® sobre comportamento saudável.

- Após a intervenção:

4) Você realizará novamente as mesmas avaliações feitas no início do projeto.

5) Caso você não atinja o resultado esperado (melhora da força/resistência muscular; melhora do condicionamento físico; melhora do nível de açúcar e de gordura no sangue; melhora na interação social), nós o encaminharemos para a Estratégia e Saúde da Família (ESF).

- É IMPORTANTE você saber que:

6) Você terá direito à indenização por eventuais danos decorrentes da pesquisa, nos termos da lei.

7) Você será informado e terá acesso aos resultados dos exames laboratoriais e resultado final da pesquisa.

Esclarecemos que ninguém saberá que você está participando da pesquisa, não falaremos a outras pessoas, nem daremos a estranhos as informações que você nos der. Os resultados da pesquisa vão ser publicados, mas sem identificar os adolescentes que participaram da pesquisa.

Esclarecemos ainda, que você não pagará e nem será remunerado(a) por sua participação. Além disso, vale ressaltar que caso haja gastos, vocês serão ressarcidos pelopesquisador.

A avaliação e o programa de atividade física são considerados seguro, mas é possível ocorrer “riscos” mínimos. Em relação a avaliação, todos os questionários serão aplicados de forma auto-relatado, ou seja, o participante responderá individualmente e de acordo com a sua própria interpretação. Assim, o adolescente NÃO será exposto ou comparado com seus colegas evitando qualquer tipo de constrangimento e transtornos psicológicos durante a avaliação. Dores musculares devido aos testes físicos, todos os testes serão conduzidos e supervisionados por pesquisadores experientes que demonstrarão aos participantes como deverá ser executado os movimentos, minimizando assim qualquer tipo de lesão por execução de movimento errado. Quanto a coleta sanguínea, os riscos também são mínimos (dores leves na hora da pulsão sanguínea), pois a coleta será realizada em local apropriado, por pessoas especializadas (enfermeiros), porém caso ocorra algum incidente o adolescente juntamente com seu responsável (que deverá estar presente no dia da coleta) serão levados imediatamente a Santa Casa de Misericórdia de Jacarezinho. Em relação a intervenção, poderá ocorrer possíveis dores musculares devido aos exercícios inseridos durante a aula de educação física e ao aumento do número de passos (intervenção da atividade física). No entanto, para aliviar e ou minimizar esse desconforto serão realizados aquecimento muscular antes de iniciar o exercício e alongamento ao final de cada sessão; também será respeitado o tempo de descanso muscular (48 horas pós atividade). Além disso, os adolescentes serão orientados quando a adotar uma alimentação saudável e hidratação diária. Caso aconteça algo errado durante a intervenção, você pode nos procurar pelos telefones ((043) 3525-0498 ou 99900-0265) da pesquisadora (Géssika Castilho dos Santos). Quanto a coleta sanguínea, os riscos também são mínimos (dores leves na hora da pulsão sanguínea), pois a coleta será realizada em local apropriado, por pessoas especializadas (enfermeiros), porém caso ocorra algum incidente você e seu responsável (que deverá estar presente no dia da coleta) serão levados imediatamente a Santa Casa de Misericórdia de Jacarezinho.

Mas há coisas boas que podem acontecer como: sem nenhum gasto, você receberá informações sobre os riscos do comportamento sedentário; participará de exercícios que irão melhorar seus músculos (aptidão muscular) e utilizará um pedômetro para auxiliar na mudança de comportamento no estilo de vida, os quais resultarão em vários benefícios: aumento do nível de atividade física dos adolescentes; redução nos níveis de açúcar e gordura no sangue, melhora dos hábitos alimentares e controle psicológico (autocontrole; automotivação; relacionamento interpessoal).

Caso você tenha dúvidas ou necessite de maiores informações sobre a pesquisa poderá nos contatar: Prof. Dr. Antonio Stabelini Neto. Centro de Ciências da Saúde da Universidade Estadual do Norte do Paraná. Alameda Padre Magno, 841 – Jacarezinho - PR - CEP 86400-000. Tel.: (043) 3525-0498. E-mail: asneto@uenp.edu.br. Prof. Me. Géssika Castilho dos Santos. Alameda Padre Magno, 841 – Jacarezinho - PR - CEP 86400-000. Tel.: (043) 3525-0498 ou (43) 99900-0265. E-mail: gessika.castilho@gmail.com.

Caso você tenha dúvidas ou denúncias de cunho ético pode procurar o Comitê de Ética (CEP/UENP, Rod. BR 369, Km 54 - Bandeirantes-PR, CEP 86360-000, Caixa Postal 261, Fone (43)3542-8056), funcionamento de segunda a sexta-feira das 7h30min às 12h e das 13h30min às 17h. Este termo deverá ser preenchido em duas vias de igual teor, sendo uma delas devidamente preenchida, assinada e entregue ao (à) voluntário(a).

Eu ___________________________________ Li e concordo em participar do estudo como voluntário.

____________________________________________

Assinatura do adolescente participante ou impressão datiloscópica

Data: ___/___/____

Eu, Antonio Stabelini Neto, declaro que forneci todas as informações referentes ao projeto de pesquisa.

____________________________________________

Antonio Stabelini Neto

Data: ___/___/____

Eu, Géssika Castilho dos Santos, declaro que forneci todas as informações referentes ao projeto de pesquisa.

____________________________________________

Géssika Castilho dos Santos

Data: ___/___/____

Jacarezinho, ___ de ________de 202_.
