# Supplementary material for: Improving physical activity behaviors, physical fitness, cardiometabolic and mental health in adolescents - ActTeens Program: A protocol for a randomized controlled trial
Source: PLoS One. 2022 Aug 9;17(8):e0272629. doi: 10.1371/journal.pone.0272629 (PMC9362910; doi:10.1371/journal.pone.0272629)
Supplement: S3 File — (DOCX) [file pone.0272629.s004.docx]

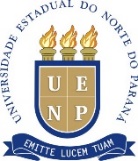
 **
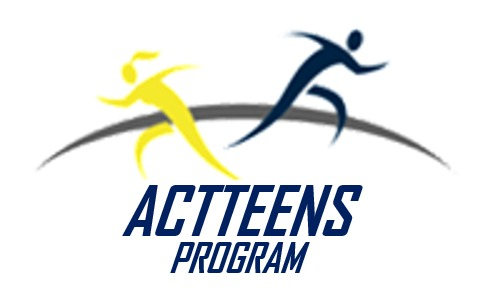

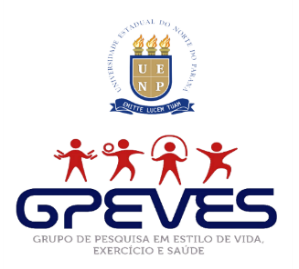
**

| Instructions for filling:  - Try to provide the requested information and indicate it by marking an “X” or filling in the spaces in the questionnaire, - Your answers will be kept confidential and the information will only be used for research purposes; - Your participation is very important! Thank you so much. |
| --- |

## **Personal Information**

**Evaluation date:** ____ /____ / _______.

## Name: ____________________________________________________________

## School: ___________________________________________ **Grade**: 8º [ ] 9º [ ]

**City:**_______________________________________________________________

Home address: _______________________________________________________

____________________________________________________________________

Cell Phone: ( )_______________________ WhatsApp: [ ] yes [ ] no

**1.** **Gender/sex:** [ ] Male [ ] Female **Birth date:** ____/____/______

**2. Study period:** [ ] in the morning – 7:20am until 12:00 pm

[ ] in the afternoon – 12:50 pm until 5:20 pm

**3. Do you live with your parents?** [ ] yes [ ] no Number of siblings: [ ____ ]

Name of the mother/father or guardian: ___________________________________

___________________________________________________________________

Telefone da mãe/pai ou responsável: ( ) _________________________________

___________________________________________________________________

**4. Live in:** [ ] rural district [ ] urban district

**5. Type of residence:** [ ] house [ ] apartment [ ] other

**6. Do you engage in any organized physical activity outside of school?**

[ ] yes [ ] no **How many times a week?**:_____________________

**SECTION 1 - SOCIOECONOMIC LEVEL**

| **WHAT IS YOUR FAMILY'S MONTHLY INCOME?** | |  |
| --- | --- | --- |
| ( ) | Family income up to one Brazilian minimum wage |  |
| ( ) | Family income up one to five Brazilian minimum wages |  |
| ( ) | Family income up five to ten Brazilian minimum wages |  |
| ( ) | Family income up 10–20 Brazilian minimum wages |  |
| ( ) | Family income up more than 20 Brazilian minimum wages |  |
| What is your **Mothers’** educational level?  ( ) Incomplete primary school  ( ) Complete primary school  ( ) Complete elementary school  ( ) Complete high school  ( ) Complete college education | | |
| What is your **Fathers’** educational level?  ( ) Incomplete primary school  ( ) Complete primary school  ( ) Complete elementary school  ( ) Complete high school  ( ) Complete college education | | |

,

**SECTION 2 – LIFESTYLE**

2.1 **EATING BEHAVIOR**

2.1.1 **Do you usually have breakfast?**

( ) Yes, every day

( ) Yes, 5 to 6 days a week

( ) Yes, 3 to 4 days a week

( ) Yes, 1 to 2 days a week

( ) No

2.1.2 **How many days in the last week have you eaten deep-fried snacks? Example: French fries or other fried snacks.**

( ) I haven’t eaten deep-fried snacks in the last 7 days

( ) Yes, 1 to 2 days a week

( ) Yes, 3 to 4 days a week

( ) Yes, 5 to 6 days a week

( ) Yes, every day

2.1.3 **How many days in the last week have you eaten** **candies (e.g. chocolate, ice cream or other)?**

( ) I haven’t eaten candies in the last 7 days

( ) Yes, 1 to 2 days a week

( ) Yes, 3 to 4 days a week

( ) Yes, 5 to 6 days a week

( ) Yes, every day

2.1.4 **How many days in the last week have you drunk soda?**

( ) I haven’t drunk soda in the last 7 days

( ) Yes, 1 to 2 days a week

( ) Yes, 3 to 4 days a week

( ) Yes, 5 to 6 days a week

( ) Yes, every day

2.1.5 **How many days in the last week have you eaten** **ultra-processed foods (e.g. hamburger, mortadella, sausage, instant noodles or packet snacks) or** **fast foods (e.g., hot dog or pizza from a fast food establishment)?**

( ) I haven’t eaten ultra-processed or fast foods in the last 7 days

( ) Yes, 1 to 2 days a week

( ) Yes, 3 to 4 days a week

( ) Yes, 5 to 6 days a week

( ) Yes, every day

**2.3 SEDENTARY BEHAVIOR**

2.3.1 **On a regular day, how much time do you spend watching television, using smartphones or computer or playing video games in your free time (except on weekends, holidays and school time)?**

( ) I don't spend time watching TV, using the smartphones or computer, or playing video games

( ) Up to 1 hour a day

( ) More than 1 hour to 2 hours a day

( ) More than 2 hours to 3 hours a day

( ) More than 3 hours to 4 hours a day

( ) More than 4 hours to 5 hours a day

( ) More than 5 hours to 6 hours a day

( ) More than 6 hours to 7 hours a day

( ) More than 7 hours

**2.4 SMOKING**

**Do you smoke?** ( ) Yes ( ) No

**2.5 ALCOHOLIC BEVERAGE**

**PLEASE NOTE:** Alcoholic beverages include: beer, wine, liquor, gin, whiskey or other

**Regardless of the amount, do you drink alcohol?** ( ) Yes ( ) No

**SECTION 3 – MENTAL HEALTH**

**3.1 - SLEEP**

| 3.1.1 **During the past month, when have you usually gone to bed at night?**  Usual bed time: _______________________________________ | | | | |
| --- | --- | --- | --- | --- |
| 3.1.2 **During the past month, how long (in minutes) has it usually take you to fall asleep each night?**  Number of minutes: _______________________________________ | | | | |
| 3.1.3 **During the past month, when have you usually gotten up in the morning?**  Usual getting up time: ____________________________________ | | | | |
| 3.1.4 **During the past month, how many hours of actual sleep did you get at night? (This may be different**  **than the number of hours you spend in bed.)** Hours of sleep per night: _____________________________ | | | | |
| 3.1.5 **During the past month, how often have you had trouble sleeping because you...** | | | | |
|  | **Not during the past month** | **Less than once a week** | **Once or twice a week** | **Three or more times a week** |
| 3.1.5.1 Cannot get to sleep within 30 minutes | ( ) | ( ) | ( ) | ( ) |
| 3.1.5.2 Wake up in the middle of the night or early morning | ( ) | ( ) | ( ) | ( ) |
| 3.1. 5.3 Have to get up to use the bathroom | ( ) | ( ) | ( ) | ( ) |
| 3.1. 5.4 Cannot breathe comfortably | ( ) | ( ) | ( ) | ( ) |
| 3.1. 5.5 Cough or snore loudly | ( ) | ( ) | ( ) | ( ) |
| 3.1.5.6 Feel too cold | ( ) | ( ) | ( ) | ( ) |
| 3.1.5.7 Feel too hot | ( ) | ( ) | ( ) | ( ) |
| 3.1.5.8 Had bad dreams | ( ) | ( ) | ( ) | ( ) |
| 3..5.9 Have pain | ( ) | ( ) | ( ) | ( ) |
| 3.1.5.10 How often during the past month have you had trouble sleeping because of this? | ( ) | ( ) | ( ) | ( ) |
| 3.1.6 **During the past month, how would you rate your sleep quality overall?**  ( ) Very good ( ) Fairly good ( ) Fairly bad ( ) Very bad | | | | |
| 3.1.7 **During the past month, how often have you taken medicine (prescribed or “over the counter”) to help**  **you sleep?**  ( ) not during the past month ( ) once or twice a week  ( ) less than once a week ( ) three or more times a week | | | | |
| 3.1.8 **During the past month, how often have you had trouble staying awake while driving, eating meals, or**  **engaging in social activity?**  ( ) not during the past month ( ) once or twice a week  ( ) less than once a week ( ) three or more times a week | | | | |
| 3.1.9 **During the past month, how much of a problem has it been for you to keep up enough enthusiasm to**  **get things done?**  ( ) no problem at all ( ) somewhat of a problem  ( ) only a very slight problem ( ) a very big problem | | | | |

**3.2 DEPRESSION, ANXIETY AND STRESS SCALE**

| Please read each statement and circle a number 0, 1, 2 or 3 which indicates how much the statement applied to you **over the past week.** There are no right or wrong answers. Do not spend too much time on any statement. | | | | | | |
| --- | --- | --- | --- | --- | --- | --- |
|  | **^0^ Did not apply to me at all** | | **^1^ Applied to me to some degree, or some of the time** | | **^2^ Applied to me to a considerable degree or a good part of time** | **^3^ Applied to me very much or most of the time** |
| I found it hard to wind down | | ( ) | | ( ) | ( ) | ( ) |
| I was aware of dryness of my mouth | | ( ) | | ( ) | ( ) | ( ) |
| I couldn’t seem to experience any positive feeling at all | | ( ) | | ( ) | ( ) | ( ) |
| I experienced breathing difficulty (e.g. excessively rapid breathing, breathlessness in the absence of physical exertion) | | ( ) | | ( ) | ( ) | ( ) |
| I found it difficult to work up the initiative to do things | | ( ) | | ( ) | ( ) | ( ) |
| I tended to over-react to situations | | ( ) | | ( ) | ( ) | ( ) |
| I experienced trembling (e.g. in the hands) | | ( ) | | ( ) | ( ) | ( ) |
| I felt that I was using a lot of nervous energy | | ( ) | | ( ) | ( ) | ( ) |
| I was worried about situations in which I might panic and make a fool of myself | | ( ) | | ( ) | ( ) | ( ) |
| I felt that I had nothing to look forward to | | ( ) | | ( ) | ( ) | ( ) |
| I found myself getting agitated | | ( ) | | ( ) | ( ) | ( ) |
| I found it difficult to relax | | ( ) | | ( ) | ( ) | ( ) |
| I felt down-hearted and blue | | ( ) | | ( ) | ( ) | ( ) |
| I was intolerant of anything that kept me from getting on with what I was doing | | ( ) | | ( ) | ( ) | ( ) |
| I felt I was close to panic | | ( ) | | ( ) | ( ) | ( ) |
| I was unable to become enthusiastic about anything | | ( ) | | ( ) | ( ) | ( ) |
| I felt I wasn’t worth much as a person | | ( ) | | ( ) | ( ) | ( ) |
| I felt that I was rather touchy | | ( ) | | ( ) | ( ) | ( ) |
| I was aware of the action of my heart in the absence of physical exertion (e.g. sense of heart rate increase, heart missing a beat) | | ( ) | | ( ) | ( ) | ( ) |
| I felt scared without any good reason | | ( ) | | ( ) | ( ) | ( ) |
| I felt that life was meaningless | | ( ) | | ( ) | ( ) | ( ) |

**3.3 PSYCHOLOGICAL WELL-BEING**

| Please read every question carefully. When you think of your answer please try to remember the **last week**. | | | | | |
| --- | --- | --- | --- | --- | --- |
|  | **not at all** | **slightly** | **moderately** | **very** | **extremely** |
| 3.4.1 Has your life been enjoyable? | **( )** | **( )** | **( )** | **( )** | **( )** |
|  | **never** | **seldom** | **quite often** | **very often** | **always** |
| 3.4.2 Have you been in a good mood? | **( )** | **( )** | **( )** | **( )** | **( )** |
| 3.4.3 Have you had fun? | **( )** | **( )** | **( )** | **( )** | **( )** |
| 3.4.4 Have you felt sad? | **( )** | **( )** | **( )** | **( )** | **( )** |
| 3.4.5 Have you felt so bad that you didn’t want to do anything? | **( )** | **( )** | **( )** | **( )** | **( )** |
| 3.4.6 Have you felt lonely? | **( )** | **( )** | **( )** | **( )** | **( )** |
| 3.4.7 Have you been happy with the way you are? | **( )** | **( )** | **( )** | **( )** | **( )** |

**SECTION 4 - CONSTRUCTS OF THE SELF-DETERMINATION THEORY**

**4.1 MOTIVATION FOR PHYSICAL ACTIVITY**

We are interested in the reasons underlying peoples’ decisions to engage, or not engage in physical activity. Using the scale below, please indicate to what extent each of the following items is true for you. Please note that there are no right or wrong answers and no trick questions. We simply want to know how you personally feel about physical activity. Your responses will be held in confidence and only used for our research purposes.

| *Why do you engage in physical activity?*....... | | | | | |
| --- | --- | --- | --- | --- | --- |
|  | Not true for me  **0** | **1** | Sometimes true for me  **2** | **3** | Sometimes true for me  **4** |
| 4.1.1 I value the benefits of physical activity | **( )** | **( )** | **( )** | **( )** | **( )** |
| 4.1.2 I do physical activity because it’s fun | **( )** | **( )** | **( )** | **( )** | **( )** |
| 4.1 3 It’s important to me to do physical activity regularly | **( )** | **( )** | **( )** | **( )** | **( )** |
| 4.1.4 I enjoy my physical activity sessions | **( )** | **( )** | **( )** | **( )** | **( )** |
| 4.1.5 I think it is important to make the effort to do physical activity regularly | **( )** | **( )** | **( )** | **( )** | **( )** |
| 4.1.6 I find physical activity a pleasurable activity | **( )** | **( )** | **( )** | **( )** | **( )** |
| 4.1.7 I get restless if I don’t do physical activity regularly | **( )** | **( )** | **( )** | **( )** | **( )** |
| 4.1.8 I get pleasure and satisfaction from participating in physical activity | **( )** | **( )** | **( )** | **( )** | **( )** |

**4.2 BASIC PSYCHOLOGICAL NEED SCALE - FRIENDS/PEERS**

| When exercising during physical education lessons..... | Strongly  Disagree | Partially  disagree | Disagree  a bit | Neither  agree nor disagree | Agree  a bit | Partially  Agree | Strongly  Agree |
| --- | --- | --- | --- | --- | --- | --- | --- |
| 4.2.1 I feel that they understand why I  choose to exercise | **( )** | **( )** | **( )** | **( )** | **( )** | **( )** | **( )** |
| 4.2.2 I feel that they encourage me to do  the exercise activities that I want to do | **( )** | **( )** | **( )** | **( )** | **( )** | **( )** | **( )** |
| 4.2.3 I feel that they listen to me about  how I would like to take part in  exercise activities | **( )** | **( )** | **( )** | **( )** | **( )** | **( )** | **( )** |
| 4.2.4 They display confidence in my  exercise ability | **( )** | **( )** | **( )** | **( )** | **( )** | **( )** | **( )** |
| 4.2.5 They help me improve my exercise  abilities | **( )** | **( )** | **( )** | **( )** | **( )** | **( )** | **( )** |
| 4.2.6 They help me to feel like I am able  to do challenging exercise activities | **( )** | **( )** | **( )** | **( )** | **( )** | **( )** | **( )** |
| 4.2.7 I feel that they care about me | **( )** | **( )** | **( )** | **( )** | **( )** | **( )** | **( )** |
| 4.2.8 I feel accepted by them | **( )** | **( )** | **( )** | **( )** | **( )** | **( )** | **( )** |
| 4.2.9 I feel that I am valued by them | **( )** | **( )** | **( )** | **( )** | **( )** | **( )** | **( )** |

**4.3 BASIC PSYCHOLOGICAL NEED SCALE - TEACHERS**

| When exercising during physical education lessons..... | Strongly  Disagree | Partially  disagree | Disagree  a bit | Neither  agree nor disagree | Agree  a bit | Partially  Agree | Strongly  Agree |
| --- | --- | --- | --- | --- | --- | --- | --- |
| 4.3.1 I feel that they understand why I  choose to exercise | **( )** | **( )** | **( )** | **( )** | **( )** | **( )** | **( )** |
| 4.3.2 I feel that they encourage me to do  the exercise activities that I want to do | **( )** | **( )** | **( )** | **( )** | **( )** | **( )** | **( )** |
| 4.3.3 I feel that they listen to me about  how I would like to take part in  exercise activities | **( )** | **( )** | **( )** | **( )** | **( )** | **( )** | **( )** |
| 4.3.4 They display confidence in my  exercise ability | **( )** | **( )** | **( )** | **( )** | **( )** | **( )** | **( )** |
| 4.3.5 They help me improve my exercise  abilities | **( )** | **( )** | **( )** | **( )** | **( )** | **( )** | **( )** |
| 4.3.6 They help me to feel like I am able  to do challenging exercise activities | **( )** | **( )** | **( )** | **( )** | **( )** | **( )** | **( )** |
| 4.3.7 I feel that they care about me | **( )** | **( )** | **( )** | **( )** | **( )** | **( )** | **( )** |
| 4.3.8 I feel accepted by them | **( )** | **( )** | **( )** | **( )** | **( )** | **( )** | **( )** |
| 4.3.9 I feel that I am valued by them | **( )** | **( )** | **( )** | **( )** | **( )** | **( )** | **( )** |

**SECTION 5 – CONSTRUCTS OF THE SOCIAL COGNITIVE** **THEORY**

**5.1 SELF-EFFICACY**

| Self-efficacy to do resistance training | | | | | |
| --- | --- | --- | --- | --- | --- |
| Confidence to do resistance training | Strongly Disagree | Disagree | Neutral | Agree | Strongly Agree |
| 5.1.1 I have the strength to complete resistance training exercises | ( ) | ( ) | ( ) | ( ) | ( ) |
| 5.1.2 I can complete resistance training exercises without the help of someone else (e.g. friend, trainer) | ( ) | ( ) | ( ) | ( ) | ( ) |
| 5.1.3 If I don’t have access to a gym I can still do resistance training (e.g. body weight exercises) | ( ) | ( ) | ( ) | ( ) | ( ) |
| 5.1.4 I have the skill and technique to complete resistance training exercises safely | ( ) | ( ) | ( ) | ( ) | ( ) |
