# Supplementary material for: Improving physical activity behaviors, physical fitness, cardiometabolic and mental health in adolescents - ActTeens Program: A protocol for a randomized controlled trial
Source: PLoS One. 2022 Aug 9;17(8):e0272629. doi: 10.1371/journal.pone.0272629 (PMC9362910; doi:10.1371/journal.pone.0272629)
Supplement: S4 File — (PDF) [file pone.0272629.s005.pdf]

ActTeens Program - Lesson Observation and Feedback

School:	Teacher:	Weather conditions:	Girls-only/Boys-only/ Mix

Term:	Date:	Time:	Participating students:	Non-participating students:

Was the teacher using the 'ActTeens' teaching resources (e.g. activity cards)?	YES	NO

Adherence to session structure *(circle responses and provide comments)*

WARM-UP	i)	Warm-up involves movement-based games	YES	NO
	ii)	Warm-up includes dynamic stretching	YES	NO
	Comments:			
FIT WORKOUT	i)	Sessions provides opportunity for students to develop muscle-strengthening exercise skills	YES	NO
	Comments:			
HIRT WORKOUT	i)	Session involves a high-intensity workout including exercise for muscle-strengthening and cardiorespiratory	YES	NO
	Comments:			
COOL DOWN	i)	Session includes static stretching	YES	NO
	ii)	Teacher discusses behavioural messages	YES	NO
	iii)	Teacher reinforces key skill components or concepts	YES	NO

General comments:

**Adherence to SAAFE teaching principles** *(circle and provide comments)*		*(1 = Not at all true to 5 = Very true)*				
SUPPORTIVE	i) Teacher provides individual skill specific feedback	1	2	3	4	5
	ii) Teacher provides feedback on student effort and involvement	1	2	3	4	5
	iii) Teacher promotes positive interactions between students	1	2	3	4	5
	Comments:					

ACTIVE	i) Activities involve small-sided games and circuits	1	2	3	4	5
	ii) Teacher monitors students' activity levels (visually or using pedometers)	1	2	3	4	5
	iii) Equipment is plentiful	1	2	3	4	5
	iv) Efficient transitions between activities	1	2	3	4	5
	Comments:					
AUTONOMOUS	i) Teacher reinforces the relevance of the activities	1	2	3	4	5
	ii) Students are given choices about the tasks and activities	1	2	3	4	5
	iii) Students are involved in the set-up and running of activities	1	2	3	4	5
	Comments:					
FAIR	i) Teacher ensures that students are evenly matched in activities	1	2	3	4	5
	ii) Teacher acknowledges and rewards good sportsmanship	1	2	3	4	5
	iii) If necessary, teacher modifies activities to maximise opportunities for success	1	2	3	4	5
	Comments:					
ENJOYABLE	i) Session starts with an enjoyable activity	1	2	3	4	5
	ii) Session finishes with an enjoyable activity	1	2	3	4	5
	iii) Session involves a wide variety of activities	1	2	3	4	5
	Comments:					
General comments:						

Name: ______________________

School: _____________________

ActTeens Program
Student end-program evaluation

Thank you for taking part in the ActTeens Program. We would like to know what you thought of the program and would be grateful if you could complete the following questionnaire.

Please be honest in your reply. All responses will be treated in confidence.

**1) Overall satisfaction:**	**Poor**	**Fair**	**Average**	**Good**	**Excellent**
a. Overall, I would rate the ActTeens program as…	☐	☐	☐	☐	☐
b. Overall, I would rate my teacher's delivery of the ActTeens program as…	☐	☐	☐	☐	☐

**2) My ActTeens teacher:**	**Strongly Disagree**	**Disagree**	**Neutral**	**Agree**	**Strongly Agree**
a. I found my teacher easy to relate to	SD	D	N	A	SA
b. I found my teacher knowledgeable about health and fitness	SD	D	N	A	SA
c. I liked the teacher that usually delivered the ActTeens practical sessions	SD	D	N	A	SA
**3) The ActTeens program:**					
a. I enjoyed participating in the ActTeens practical sessions	SD	D	N	A	SA
b. The presentation delivered by my teacher at the beginning of the program provided me with useful information about living a healthy lifestyle	SD	D	N	A	SA

PLEASE TURN OVER

4) **WhatsApp:**	**Never**	**Rarely**	**Sometimes**	**Often**
I used the group's ActTeens in WhatsApp…	N	R	S	O
I have my own smartphone		Yes	No	

5) **ActTeens group in WhatsApp:**	**Strongly Disagree**	**Disagree**	**Neutral**	**Agree**	**Strongly Agree**
a. The messages sent through the WhatsApp reminded me to be more active, reduce my screen-time, drink less sugary drinks and limit my 'sometimes' foods	SD	D	N	A	SA
b. I enjoyed using the group to talk with my peers and exchange information, and strategies on how to be more active.	SD	D	N	A	SA
c. I enjoyed using the group to help achieve my steps-per-day goals for increasing my physical activity.	SD	D	N	A	SA

6) **Pedometer:**	**Strongly Disagree**	**Disagree**	**Neutral**	**Agree**	**Strongly Agree**
a. Feedback provided by the pedometer motivated/helped me to be more active.	SD	D	N	A	SA
b. By using the pedometer daily, I changed my behavior to increase the number of steps/day	SD	D	N	A	SA
c. The setting-goals were easy to achieve	SD	D	N	A	SA

ACTTEENS
PROGRAM

7) What did you like about the ActTeens program?

____

____

____

8) Do you have any suggestions to improve the ActTeens program?

____

____

____
